# The development of an ingestible biosensor for the characterization of gut metabolites related to major depressive disorder: hypothesis and theory

**DOI:** 10.3389/fsysb.2023.1274184

**Published:** 2023-12-05

**Authors:** Amanda Densil, Mya Elisabeth George, Hala Mahdi, Andrew Chami, Alyssa Mark, Chantal Luo, Yifan Wang, Aribah Ali, Pengpeng Tang, Audrey Yihui Dong, Sin Yu Pao, Rubani Singh Suri, Isabella Valentini, Lila Al-Arabi, Fanxiao Liu, Alesha Singh, Linda Wu, Helen Peng, Anjana Sudharshan, Zoha Naqvi, Jayda Hewitt, Catherine Andary, Vincent Leung, Paul Forsythe, Jianping Xu

**Affiliations:** ^1^ Faculty of Health Sciences, McMaster University, Hamilton, ON, Canada; ^2^ Faculty of Engineering, McMaster University, Hamilton, ON, Canada; ^3^ Faculty of Humanities, McMaster University, Hamilton, ON, Canada; ^4^ Faculty of Science, McMaster University, Hamilton, ON, Canada; ^5^ W Booth School of Engineering Practice and Technology, McMaster University, Hamilton, ON, Canada; ^6^ Arts and Science Program, McMaster University, Hamilton, ON, Canada; ^7^ Department of Chemical Engineering, Faculty of Engineering, McMaster University, Hamilton, ON, Canada; ^8^ Division of Pulmonary Medicine, Department of Medicine, Faculty of Medicine and Dentistry, Alberta Respiratory Centre, University of Alberta, Edmonton, AB, Canada; ^9^ Department of Biology, Faculty of Science, McMaster University, Hamilton, ON, Canada

**Keywords:** major depressive disorder, ingestible biosensor, gut brain axis, gene editing, gut microbiome, personalized healthcare

## Abstract

The diagnostic process for psychiatric conditions is guided by the Diagnostic and Statistical Manual of Mental Disorders (DSM) in North America. Revisions of the DSM over the years have led to lowered diagnostic thresholds across the board, incurring increased rates of both misdiagnosis and over-diagnosis. Coupled with stigma, this ambiguity and lack of consistency exacerbates the challenges that clinicians and scientists face in the clinical assessment and research of mood disorders such as Major Depressive Disorder (MDD). While current efforts to characterize MDD have largely focused on qualitative approaches, the broad variations in physiological traits, such as those found in the gut, suggest the immense potential of using biomarkers to provide a quantitative and objective assessment. Here, we propose the development of a probiotic *Escherichia coli* (*E. coli*) multi-input ingestible biosensor for the characterization of key gut metabolites implicated in MDD. DNA writing with CRISPR based editors allows for the molecular recording of signals while riboflavin detection acts as a means to establish temporal and spatial specificity for the large intestine. We test the feasibility of this approach through kinetic modeling of the system which demonstrates targeted sensing and robust recording of metabolites within the large intestine in a time- and dose- dependent manner. Additionally, a post-hoc normalization model successfully controlled for confounding factors such as individual variation in riboflavin concentrations, producing a linear relationship between actual and predicted metabolite concentrations. We also highlight indole, butyrate, tetrahydrofolate, hydrogen peroxide, and tetrathionate as key gut metabolites that have the potential to direct our proposed biosensor specifically for MDD. Ultimately, our proposed biosensor has the potential to allow for a greater understanding of disease pathophysiology, assessment, and treatment response for many mood disorders.

## 1 Introduction

Personalized medicine has emerged as a novel model in recent years for patient profiling, taking into account an individuals’ predicted response or risk to a disease and curating a specific diagnosis and individualized treatment, as opposed to prescribing a one-size-fits all treatment plan. For example, gathering biometric data to serve as a baseline for an individual, aids in determining the critical point at which a specific treatment is needed, instead of using generalized ranges and guidelines designed to fit an entire population. Thus, these interventions are more efficacious and cost-effective at reducing treatment failure rate (Ginsburg et al., 2018; König et al., 2017).

A personalized-medicine based approach is applicable for mood disorders such as major depressive disorder (MDD) as the literature details high individual-to-individual variability, resulting in variable treatment response and success rates (Knudsen et al., 2021). Ultimately, our research aims to significantly improve patient outcomes by improving the accuracy of MDD diagnosis, leading to more informed treatment plans that can ultimately minimize the financial, social, and environmental costs associated with this condition.

**FIGURE 1:**
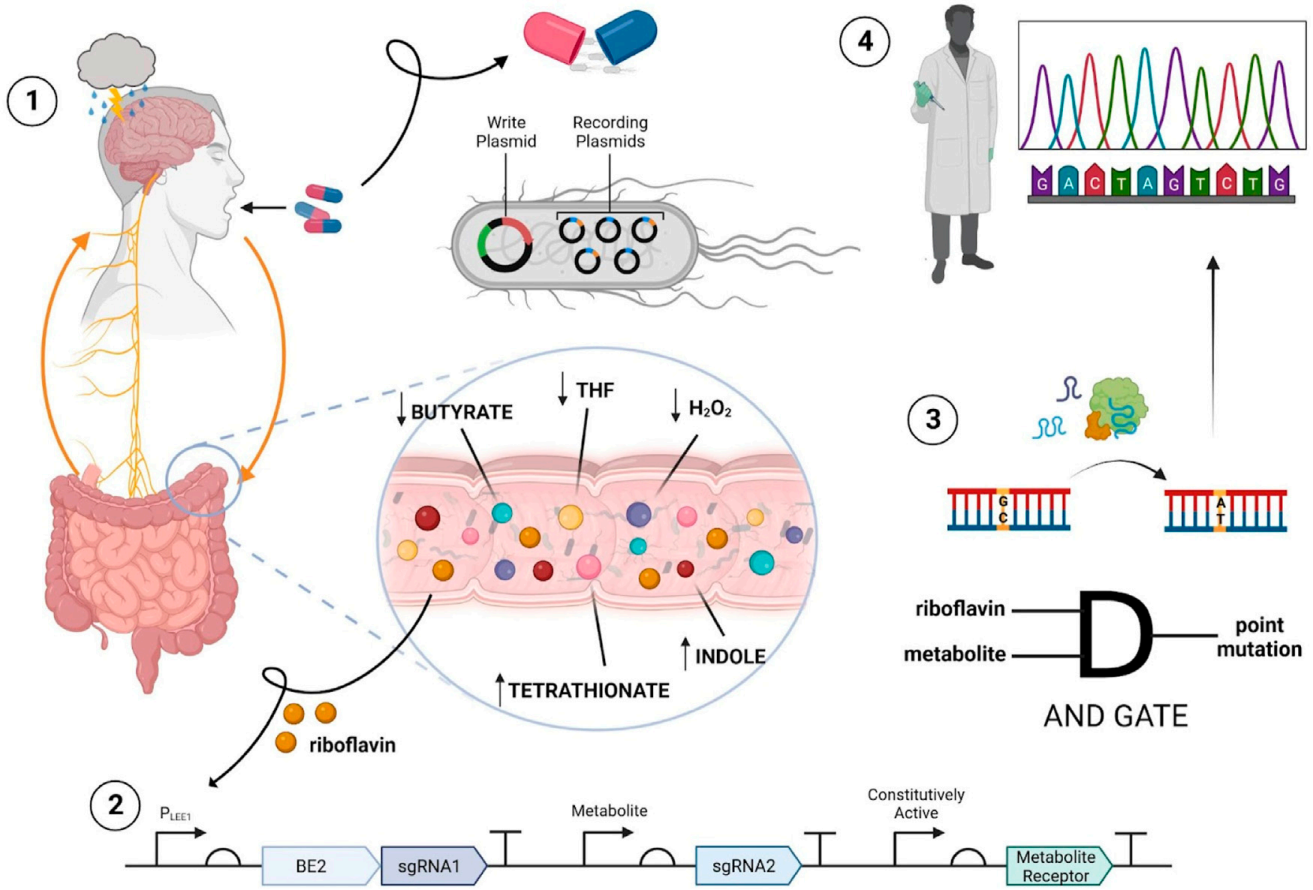
Overview of proposed ingestible biosensor system. The process begins with oral administration of the encapsulated biosensor containing engineered bacteria to an individual with MDD (step 1). Upon ingestion, direct surveillance and recording of target gut metabolites can occur in the large intestine due to riboflavin specificity (step 2). Leveraging AND gate functionality allows for BE2, sgRNAs and the metabolite receptor sensing systems to be transcribed, and for base editing to create point mutations in the recording plasmids (step 3). After excretion of the biosensor, the encoded mutations are subject to analysis via sequencing to extract information concerning target metabolite concentrations in relation to MDD (step 4).

Here, we propose a tool that is geared towards researchers studying the gut-brain axis (GBA) in the context of psychiatric and gastrointestinal (GI) diseases. Our ingestible biosensor allows for the direct measurement and molecular recording of gut biomarker concentrations, with spatial and temporal specificity for desired regions of the GI tract. The recorded data within the bacterial system can then easily be extracted after excretion, using standard DNA sequencing protocols (Figure 1). Notably, there is immense potential for the system to be tailored and adapted for any disease of interest concerning dysbiosis and the GBA.

### 1.1 Ingestible biosensors and their potential

Biosensors are state-of-the-art, compact, analytical devices that can detect and measure levels of specific biological molecules indicative of a disease or condition, otherwise known as a biomarker (Naresh and Lee, 2021). Engineered biosensors can selectively detect biomarkers associated with alterations in biological processes and convert biological signals into an electrical, chemical, or biological output (Bhalla et al., 2016).

Ingestible biosensors have recently emerged in academia and in clinical settings as a method of monitoring the gut environment and microbiota *in situ* and in real time (Chai et al., 2016; Pan and Wang, 2012; Thomas, 2018). One microbial electronic biosensor was created by Mimee et al. (2018) to allow for *in situ* biomolecular detection of gastric bleeding. The design included a probiotic *Escherichia coli* strain engineered to release a bioluminescent reporter in response to the detection of liberated heme from lysed red blood cells; electronic circuits in the capsule were designed to detect this luminescence and wirelessly communicate the results to an external device. Alternatively, the MyTMed capsule was designed to monitor medication adherence, by sensing the release of medication through the dissolution of a gelatin capsule and generating a unique radiofrequency signal to be broadcasted to a wearable receiver (Chai et al., 2016). It is important to note, however, that the electronic components of certain biosensors have caused concerns surrounding data security (Gerke et al., 2019) which we hope to mitigate through the creation of a purely biological sensing mechanism.

#### 1.1.1 Exploiting the gut-brain axis using an ingestible biosensor to investigate mood disorders

The GBA refers to the bidirectional pathway between the enteric nervous system (ENS) and the central nervous system (CNS), relating neural functions in the brain to intestinal functions in the gut and *vice versa* (Carabotti et al., 2015). It has been postulated that this connection may be critical to the investigation of mood disorder pathogenesis and treatment, a traditionally neuro-centered field of research, as imbalances in gut microbiota (dysbiosis) have been found to contribute to the development of various mood disorders such as generalized anxiety disorder, chronic stress, and MDD (Carabotti et al., 2015; Caspani et al., 2019).

Current monitoring and diagnostic efforts of the GI tract are primarily focused on fecal metabolomics. This method is noninvasive, inexpensive, and repeatable, however, multiple studies have shown discrepancies in the accuracy of using fecal samples as a proxy for mucosal microbiota representation (Rangel et al., 2015; Ringel et al., 2015; Tap et al., 2017). On the other hand, sampling directly from the GI tract as is achieved with endoscopic biopsies, is a more reliable and accurate method of characterizing this unique environment. However, this procedure poses the issue of extreme invasiveness which results in low patient compliance (Nagata et al., 2019). As such, present methods involve significant drawbacks and ultimately do not provide an accurate enough reflection of the composition of the gut microbiota (Tang et al., 2020).

Ingestible biosensors mitigate many of these concerns as they are relatively non-invasive, patient-friendly, and easily repeatable, all while still sampling directly from the GI tract. By monitoring internal molecular components, ingestible biosensors have the potential to equip researchers with a more accurate way of investigating the GBA, providing a means for early detection of diseases that have not yet externally manifested, or providing confirmation of disorders that are currently measured by qualitative methods such as mood disorders (Haleem et al., 2021).

### 1.2 Major depressive disorder

MDD is a prevalent mental health condition that poses significant challenges for diagnosis and treatment. Its etiology is multifaceted, with biological, genetic, environmental, and social factors all contributing to the onset of depressive symptoms. The Social Signal Transduction Theory of Depression proposes that the combination of genetic predisposition alongside social stressors such as conflict, isolation, and exclusion, result in an individual developing depression (Slavich and Irwin, 2014; Slavich and Sacher, 2019).

#### 1.2.1 The qualitative nature of MDD diagnosis

According to the Diagnostic and Statistical Manual of Mental Disorders, Fifth Edition (DSM-5), in order to be diagnosed with MDD, a patient needs to experience five or more depressive symptoms for more than 2 weeks; these symptoms must impair an individual’s ability to function on a daily basis, conduct their typical work and navigate relational and educational pursuits (Table 1). As such, diagnosis is primarily qualitative, heavily relying on patient reporting and physician comprehension. This results in subjective symptomatology, especially as the professional understanding and categorization of MDD has changed over time. In 2013, the DSM-IV transitioned to become the DSM-V, which introduced many changes such as discrimination between single and recurrent depressive episodes (drawing more attention to persistent episodes) and acknowledged that bereavement can potentially lead to symptoms characteristic of MDD (as first seen in the DSM-IV-TR when the DSM-IV excluded bereavement from depressive disorders) (Table 1). These alterations in diagnostic thresholds can lead to over- and or misdiagnosis in MDD, and lack consistency which is needed for accurate diagnosis.

**TABLE 1:** DSM-IV to DSM-V major depressive disorder comparison.

DSM edition	Class	Symptoms
DSM-IV	Mood Disorders	A1) Depressed mood or irritability
		A2) Diminished interest in daily activities
DSM-V	Depressive Disorders	A3) Significant weight gain or loss
		A4) Insomnia
		A5) Agitation
		A6) Fatigue or loss of energy
		A7) Feeling worthless or inappropriate guilt
		A8) Diminished ability to concentrate
		A9) Thoughts of suicide, plan or attempt in death

Criteria (both DSM-IV and DSM-V): 5 or more symptoms for more than 2 weeks; during the 2 week period, must experience depressed mood daily, symptoms must intervene with daily activities, and no manic or hypomanic behavior. Criteria (DSM-IV only): Symptoms not related to mixed episode including manic and depressive characteristics, symptoms not better accounted for by bereavement. Source for Table 1: (O’Connor, 2009; Lyngzeidetson, 2014; Substance Abuse and Mental Health Services Administration, 2016).

#### 1.2.2 The impact and disease burden of MDD and why accurate diagnosis is needed

The number of individuals diagnosed with MDD has seen a huge increase since 2005; especially in the past 3 years, a greater disease burden has been observed among adolescents and young adults (Proudman et al., 2021). Many factors have been linked to the increased prevalence of the condition including: greater awareness and education of mental health, growing economic concerns in different regions, and the widely experienced reality of social isolation and limited access to healthcare during the COVID-19 pandemic.

Lockdown and other public health recommendations in place from 2020 to 2022, have had a tremendous impact on peoples of all ages, with feelings of loneliness and isolation being a common finding. Results from a cross-sectional online survey distributed in 101 countries indicated that 21% of respondents experienced severe and debilitating loneliness during the pandemic, correlating to a 28% increase in the number of individuals affected by MDD in a single calendar year (O’Sullivan et al., 2021; World Health Organization, 2022a; World Health Organization, 2022b).

However, the burden of depression extends beyond an individual’s experience of the condition, as it can shape the lives of those in their community and social circles. In Canada, for example, it is estimated that the sole burden of mental illnesses and addictions in Ontario is more than 1.5 times that of all cancers, including the combined local burden of breast, colorectal, lung, and prostate cancers (Ratnasingham et al., 2013).

Depressive mood disorders have also been reported as one of the leading causes of disability worldwide, costing billions of dollars per year through losses in productivity during working hours and associated healthcare expenses. Canadians living with anxiety and depression can face unemployment rates of up to 30%, which is further hindered by challenges to receive an accurate diagnosis and have access to necessary health services (‘Unemployment, Mental Health and Substance Use’, 2014). According to the World Health Organization, depression is also a major contributor to suicide across the globe (World Health Organization, 2017), however, there remains serious gaps in appropriate support, treatment, and funding of such programs.

Thus, there is a need to complement current qualitative methods of MDD diagnosis, with a more objective and quantitative approach. This has the potential to improve diagnosis accuracy and gain a better understanding of the disorder physiologically, feeding into more informed and personalized treatment plans.

#### 1.2.3 Pathogenesis of MDD in relation to the GBA

**FIGURE 2:**
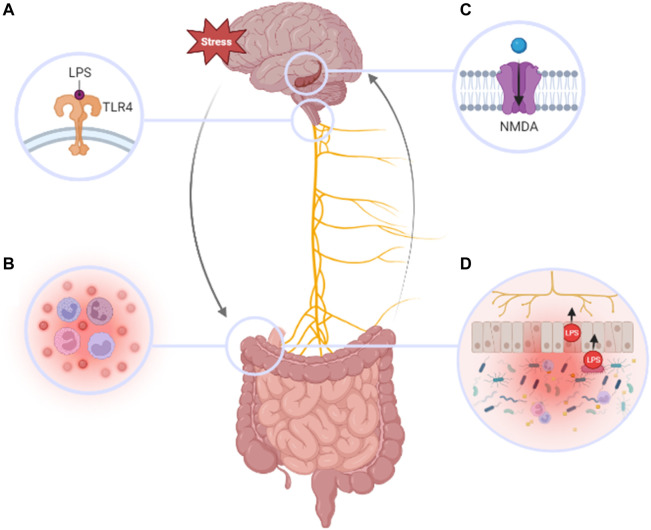
Pathogenesis of MDD in relation to the gut brain axis. Chronic stress can result in the dysregulation of several pathways that to lead to MDD including: **(A)** LPS binding to TLR-4 on the nodose ganglion, **(B)** inflammation of peripheral tissues and increased activity of pro-inflammatory cytokines and immune cells, **(C)** LPS-induced dysregulation of hippocampal BDNF and neuronal signaling by NMDA-receptors **(D)** disruption of IEB integrity, translocation of LPS, and elevated pathogenic bacterial concentrations in the gut allowing. Furthering a devastating cycle that exacerbates the severity and persistence of MDD.

There exists in literature, a clear link between MDD and the GBA (Figure 2). Taxonomic association studies in humans have identified a high prevalence of microbiome dysbiosis in MDD patients compared to healthy individuals (Ressler and Mayberg, 2007; Naseribafrouei et al., 2014; Jiang et al., 2015; Zheng et al., 2016; Caspani et al., 2019), and numerous studies using germ-free mice have also revealed the detrimental effects of uncolonized and dysbiotic gut microbiomes (Clarke et al., 2013; Luczynski et al., 2016; Matcovitch-Natan et al., 2016). One study by J. Zhang et al. (2020) demonstrated that depression-like behaviors can be induced in germ-free mice who have received transplantation of microbiota derived from MDD patients.

While the pathophysiology of MDD in relation to the GBA is not fully elucidated, the vagus nerve has been highlighted as a critical player in facilitating the effect of neuroactive gut metabolites on ENS development and CNS signaling (Braniste et al., 2014; Erny et al., 2015; Ogbonnaya et al., 2015; Morais et al., 2021). Additionally, the GBA relies on proper functioning of the blood-brain barrier (BBB) and the intestinal epithelial barrier (IEB), which are both held together by tight junction proteins, and is highly sensitive to inflammatory cytokines and endotoxins (Geng et al., 2020). Consequently, endotoxins that permeate through the IEB via the vagal afferent nerve, can disrupt neuronal signaling pathways within the brain.

##### 1.2.3.1 LPS, the vagus nerve, and BDNF

Lipopolysaccharide (LPS), a bacterial product formed from the outer membrane of Gram-negative species, is one notable endotoxin that has been reported at higher concentrations in participants with DSM-IV diagnosed MDD (Ulevitch and Tobias, 1995). In fact, administration of LPS in healthy human participants was found to induce mild depressive and cognitive symptoms via the nodose ganglion and afferent vagus nerve (Howland, 2014; Kiecolt-Glaser et al., 2018; Ulevitch and Tobias, 1995; Zhang, J. et al., 2020). LPS is suggested to act on toll-like receptor 4 (TLR-4) on the nodose ganglion, inducing an innate immune response that leads to the subsequent cascade of proinflammatory signaling molecules such as IL-1β, IL-6, IL-18, IL-22, and TNF (Kiecolt-Glaser et al., 2018). The cellular mechanisms of LPS on the vagus nerve and the brain are unclear, however, it is suggested that brain derived neurotrophic factor (BDNF) levels are significantly influenced by LPS. A study by Guan and Fang (2006) found that LPS induction resulted in a 20% decrease in hippocampal BDNF, and a 19%–63% decrease in other brain regions. On the other hand, direct stimulation of the vagus nerve in rats seemed to significantly increase BDNF mRNA concentration in the hippocampus and cerebral cortex by 26.3% and 58.1% respectively (Follesa et al., 2007). Therefore, stress and mutations, or even a deficient vagus nerve stimulation can lead to decreased or dysfunctional BDNF.

Hippocampal neurons stimulated with BDNF have also been correlated to the upregulation of NMDA subunits (NR1, NR2A and NR2B) in the plasma membrane (Caldeira et al., 2007). This results in increased NMDA receptor activation which is observed to mediate LPS-induced depressive-like behavior and impair long-term potentiation (LTP) within the hippocampus (Caldeira et al., 2007; Walker et al., 2013). LTP impairment can subsequently have detrimental effects on synaptic plasticity, memory, and learning, all of which are observed to be dysregulated in MDD patients (Martin and Finsterwald, 2011; Pittenger, 2013; O’Leary et al., 2018; Yang et al., 2020; Parrott et al., 2021).

##### 1.2.3.2 Stress, inflammation, and MDD pathogenesis

Psychological stress has also been shown to increase incidence levels of depression via elevated cortisol and adrenocorticotropic hormone secretion; this can inhibit BDNF production and therefore decrease synaptic plasticity and memory (Sudo et al., 2004; Neufeld et al., 2011; Westfall et al., 2021). The inflammatory mechanisms of chronic stress can modify gut microbial health and epithelial permeability (Sudo et al., 2004; Neufeld et al., 2011; Westfall et al., 2021). Specifically, prolonged stress stimulates the sympathetic nervous system, leading to increased catecholamine production; this has been correlated to elevated pathogenic bacterial concentrations by 10,000-fold (Sudo et al., 2004; Neufeld et al., 2011; Westfall et al., 2021). In a study conducted using mouse models, 28 days of stress-induced stimulation led to elevated levels of norepinephrine followed by significant differences in the concentration of 36 genera of bacteria (Geng et al., 2020).

A study by Bharwani et al. (2016), induced chronic social defeat in mice models found lower efficacy of synthetic and metabolic neurotransmitter precursor pathways such as the serotonin-tryptophan pathway, and reduced functional alpha-diversity of gut microbiota. They also found elevated IL-6 levels and suppressed anti-inflammatory IL-10 CD4^+^ CD25^+^ T cells in spleens. IL-6 is found at first-wave inflammatory sites and its increase is directly correlated with the reduction of microbiota abundance and blooming of pro-inflammatory genera (Bailey et al., 2011; Tanaka et al., 2014). A recent study on acute stress examined the mobilization of neutrophils and various cytokines in peripheral gut tissue (Poller et al., 2022). Specifically, in response to gut inflammation, neutrophils release chemoattractants and cytokines, which can cross the epithelium into the intestinal microbiome (Fournier and Parkos, 2012; Poller et al., 2022) where cytokines such as IL-6 may then disrupt microbial homeostasis (Bailey et al., 2011). Thus, this supports a larger, complex interplay of immune mechanisms involved in prolonged gut inflammation.

## 2 Hypothesis and rationale

In this section, we propose the following: a clinical vision for implementation of our ingestible biosensor, a design for the internal molecular recording system capable of quantifying gut metabolites with temporal and spatial specificity, a pill design to house and protect the internal components as it traverses the GI tract, and a panel of metabolites to adapt this tool specifically for MDD.

### 2.1 Proposed clinical implementation

We currently foresee the use of an ingestible biosensor as a data mining tool within the parameters of a highly controlled clinical research setting. To this effect, the target audience for this tool includes researchers interested in the GBA, who require *in vivo* data collection of gut metabolites. The implementation of such a tool could be envisioned as the following:

1. **Attain informed consent**. Ensuring that all patients have the capacity and desire to consent to the use of the ingestible biosensor within the limitations of the research study is important and is expected to be a key consideration of any study ethics approval and disclosure to participants.
2. **Assessment of suitability**. This is a critical safety step prior to administering the ingestible biosensor to ensure that the subject’s gut is in a state that can readily accept and pass the biosensor through the GI tract. This may include performing a barium X-ray in order to characterize the small and large bowel wall lining, size, shape, contour, and patency, thereby checking for bowel obstruction which would exclude the individual from the study (Palmer et al., 2011).
3. **Administration of biosensor**. Standard protocols such as special liquid diets and administration of a laxative prior to ingestion of the biosensor prepare the subject’s body for clean sampling and measurements (Hermoso-Durán et al., 2023).
4. **In-patient monitoring**. For the duration between ingestion and excretion (approximately 10 h), the subject will be monitored for signs of GI discomfort including bloating, acid reflux, abdominal pain.
5. **Collection and sequencing**. After bowel movement, the biosensor pill will be recovered and brought to an associated lab. Bacteria from the individual compartments will be isolated and cultured for independent growth of each metabolite sensing system. Based on our proposed internal recording design described in Section 2.2, metabolite sensing is reported in the form of point mutations on recording plasmids. Thus, researchers will be able to sequence the recording plasmids and analyze the proportion of edited to unedited plasmids at a specific site to calculate the metabolite concentration.
6. **Post-collection monitoring**. After collection, symptoms of discomfort, diarrhea, constipation, or any bleeding will be monitored (Lee, Erdogan, and Rao, 2014; Almario et al., 2018). Should any discomfort persist after any participant expels the biosensor pill, the participant would be connected with the appropriate medical care. Maintaining remote communication with study participants until they have had a subsequent bowel movement is also recommended.

### 2.2 Proposed internal molecular recording system

#### 2.2.1 CRISPR-mediated recording to encode metabolite concentration

Reporter systems are coding regions that can be linked to a promoter and subsequently produce outputs that are clearly distinguishable from the background activity of a cell (Li et al., 2018). They are meant to provide a means of measuring the promoter activity, and by extension, molecular species or cell pathways which regulate that promoter. With the emergence of DNA writing and sequencing technologies, researchers have developed long-term molecular recording platforms with genetic based reporters in living cells which hold great potential to serve as a reporter system for our ingestible sensor.

SCRIBE (Synthetic Cellular Recorders Integrating Biological Events), a strategy developed by Farzadfard and Lu (2014), generates intracellularly expressed single-stranded DNA (ssDNA) in response to various regulatory signals. The ssDNAs are recombined into genomic DNA at specific loci by co-expressed recombinases, resulting in precise point mutations which accumulate as a function of signal magnitude and duration. However, due to its limited recombination rate, SCRIBE requires actively dividing cells to achieve population-memory; thus, the platform is more suitable for recording across collective genomes of cellular populations, rather than editing at the single-cell level.

To overcome this dependence on large cell populations, Tang and Liu (2018) developed two CRISPR-mediated analog multi-event recording apparatus (CAMERA) systems, enabling analog recording within each cell. The systems use the CRISPR-Cas9 nuclease and Cas9-derived base editors, which are expressed on writing plasmids in response to stimuli of interest, to generate permanent DNA sequence modifications at guide RNA-specific loci on recording plasmids. Both systems achieved stable recording of the amplitude or duration of various signals; this can be extended to record multiple metabolite concentrations through assignment of associated mutations to unique locations on the plasmid. The ratio of edited to unedited DNA on these recording plasmids were shown to accurately record the duration and intensity of both chemical and light-based stimuli.

The DNA-based Ordered Memory and Iteration Network Operator (DOMINO) is another genetic based recording platform that uses CRISPR base-editing technology to manipulate DNA in response to signals of interest (Farzadfard et al., 2019). By linking the guide RNA and the Cas9 complex to separate inducible promoters, the system can be controlled by an operational signal and another independent input, thereby forming a DOMINO operator that can only generate mutations when both inducers are present.

##### 2.2.1.1 Applicability to ingestible biosensor design

The use of these genetic recording methods holds great potential for ingestible biosensing systems for several reasons. First, genetic readouts are more robust against temporal degradation; unlike fluorescent markers which have limited longevity due to protein degradation, base editing outputs remain constant for much longer (Corish and Tyler-Smith, 1999). Thus, the relationship between the initial stimulus intensity and output reading is not confounded by the time at which the output is measured. Second, CAMERA was shown to be functional across a range of conditions; it achieved recording multiple stimuli in both bacteria and mammalian cells, and maintained reliable readouts to multicopy plasmids in samples ranging from 10 to 100 cells (Tang and Liu, 2018). Therefore, base editing ratios remain stable after cell death and are passed down through bacterial generations.

**FIGURE 3:**
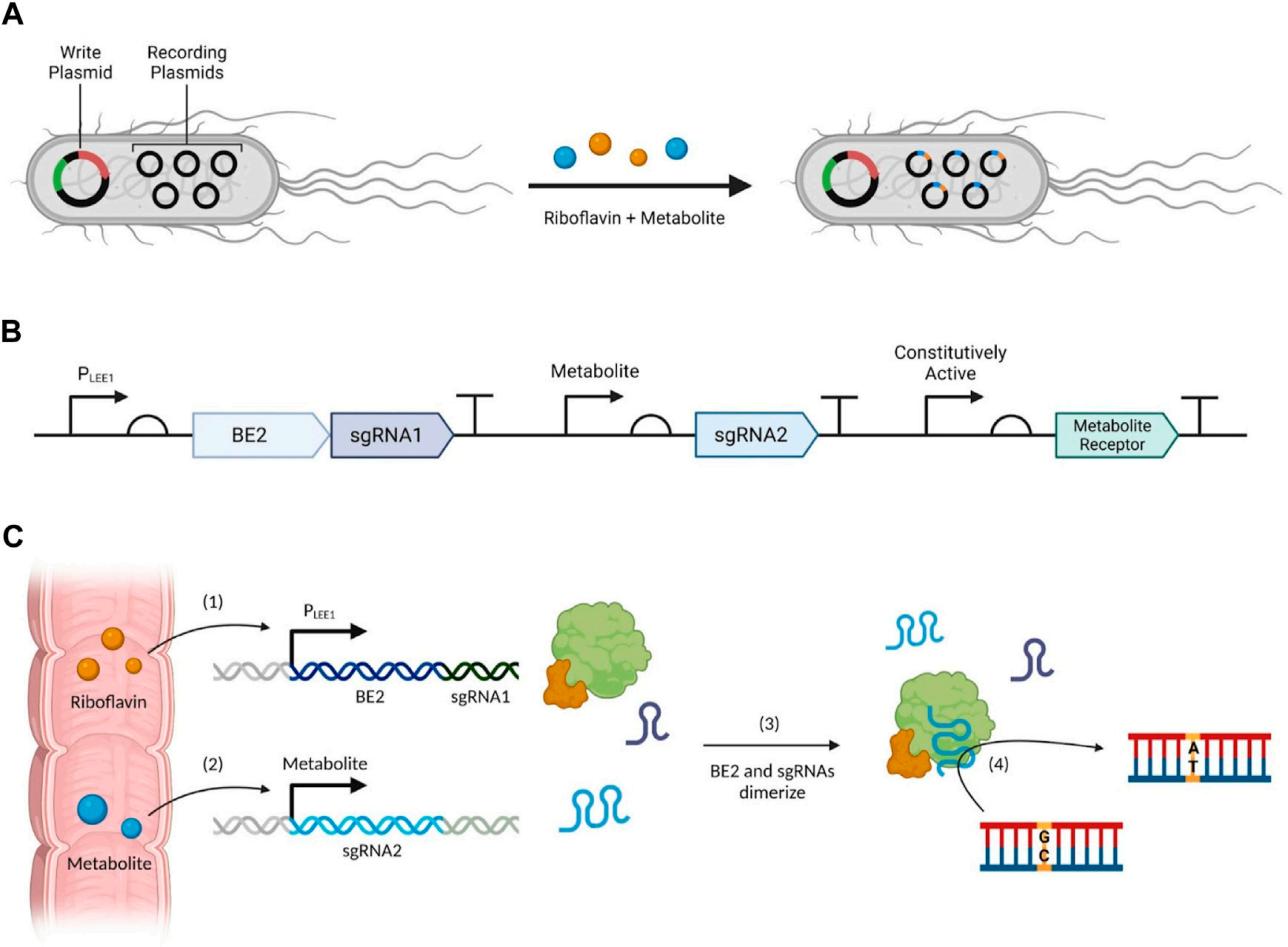
Proposed biosensor system design for the molecular reading and recording of metabolites. **(A)** An overview of the system as a whole, **(B)** the plasmid design for the LEE1 promoter (PLEE1), metabolite promoter, and the constitutively active promoter which transcribes the individual metabolite receptor sensing systems, **(C)** a flowchart of important events: (1) riboflavin induces the production of BE2 and sgRNA1 upon entry to the large intestine, and simultaneously, (2) the presence of a metabolite of interest induces the production of sgRNA2, then (3) the sgRNAs can dimerize with BE2 to (4) induce gene editing via point mutations.

Based on this previous work, we propose a genetic reporter system with *E coli*. as the chassis (Figure 3A). The system utilizes a second-generation base-editor (BE2) composed of a catalytically dead Cas9 protein (dCas9) which cannot cleave DNA fused to a cytidine deaminase (CDA); Cas9 is directed to specific DNA targets, where CDA induces a CG to AT mutation. Together, they enable programmable editing of a specific base with no detectable off-target editing, and minimal formation of indel mutations (Komor et al., 2016; Tang and Liu, 2018; Luo et al., 2020).

The dCas9 relies on single guide RNA (sgRNA) to precisely target editing sites on specialized recording plasmids, which function only to record mutations (Komor et al., 2016). These mutations are recorded within an enhanced green fluorescent protein (eGFP) gene at specific sites. The eGFP gene used contains a premature stop codon that prevents the production of the protein to mitigate redundant expression of eGFP (Tang and Liu, 2018). BE2 also includes a uracil-DNA glycosylase inhibitor (ugi) to potently block cellular repair machinery which may undo base editing (Farzadfard et al., 2019; Volke et al., 2022). The expression of these two components will be linked to two separate promoters: riboflavin will induce the expression of BE2 through the LEE1 promoter (PLEE1), while a metabolite of interest will induce the expression of the sgRNA designed by Tang and Liu (2018), sgRNA2 (Figures 3B, C). As shown in Figure 3C, once both BE2 and the sgRNA2 have been produced, they will form a complex and initiate base editing at position 186 of the eGFP gene (Komor et al., 2016; Tang and Liu, 2018). Finally, PLEE1 will also induce the expression of a separate sgRNA, sgRNA1, also designed by Tang and Liu (2018), thereby inducing a separate mutation at position 166 of the eGFP gene. The rationale for these design choices will be explained in detail in the subsequent sections.

#### 2.2.2 Targeted sensing of metabolites within the large intestine can Be achieved through riboflavin-induced activation of the LEE1 promoter

We aim to achieve targeted sensing of the large intestine for two main reasons: 1) the metabolites we aim to study have been predominantly characterized in fecal samples, which are generally considered a proxy for large intestine conditions due to their close proximity (Gevers et al., 2014), and 2) bacterial populations in the large intestine have greater numbers, increased diversity, and more stability than the small intestine (Kastl et al., 2019).

##### 2.2.2.1 Riboflavin is a large intestine-specific compound

Vitamin B2, also known as riboflavin, was identified as a compound with high specificity in the large intestine. It directly promotes the maturation of the immune system in infants, serves as an antioxidant, and is necessary for normal development, lactation, physical performance, and reproduction (Mahabadi et al., 2023). Approximately 60% of commensal bacterial species in the large intestine of humans produce riboflavin, including eight probiotic strains from five different probiotic formulations containing *Bacillus* clausii, B. subtilis, B. cereus IP 5832, and L. rhamnosus ATCC 53103 (Thakur et al., 2016; Liu et al., 2022). Although riboflavin can also be acquired through the diet, it is rapidly absorbed in the proximal part of the small intestine. As demonstrated by a mouse study by Liu et al. (2022), the concentration of naturally-occurring riboflavin in the colon and the ileum was 22.8 μM and 0.2 μM, respectively. Thus, the utilization of riboflavin as an inducer allows our proposed sensing system to be specific to the large intestine.

##### 2.2.2.2 Activation of the two-component sensing system by riboflavin to induce the LEE1 promoter

**FIGURE 4:**
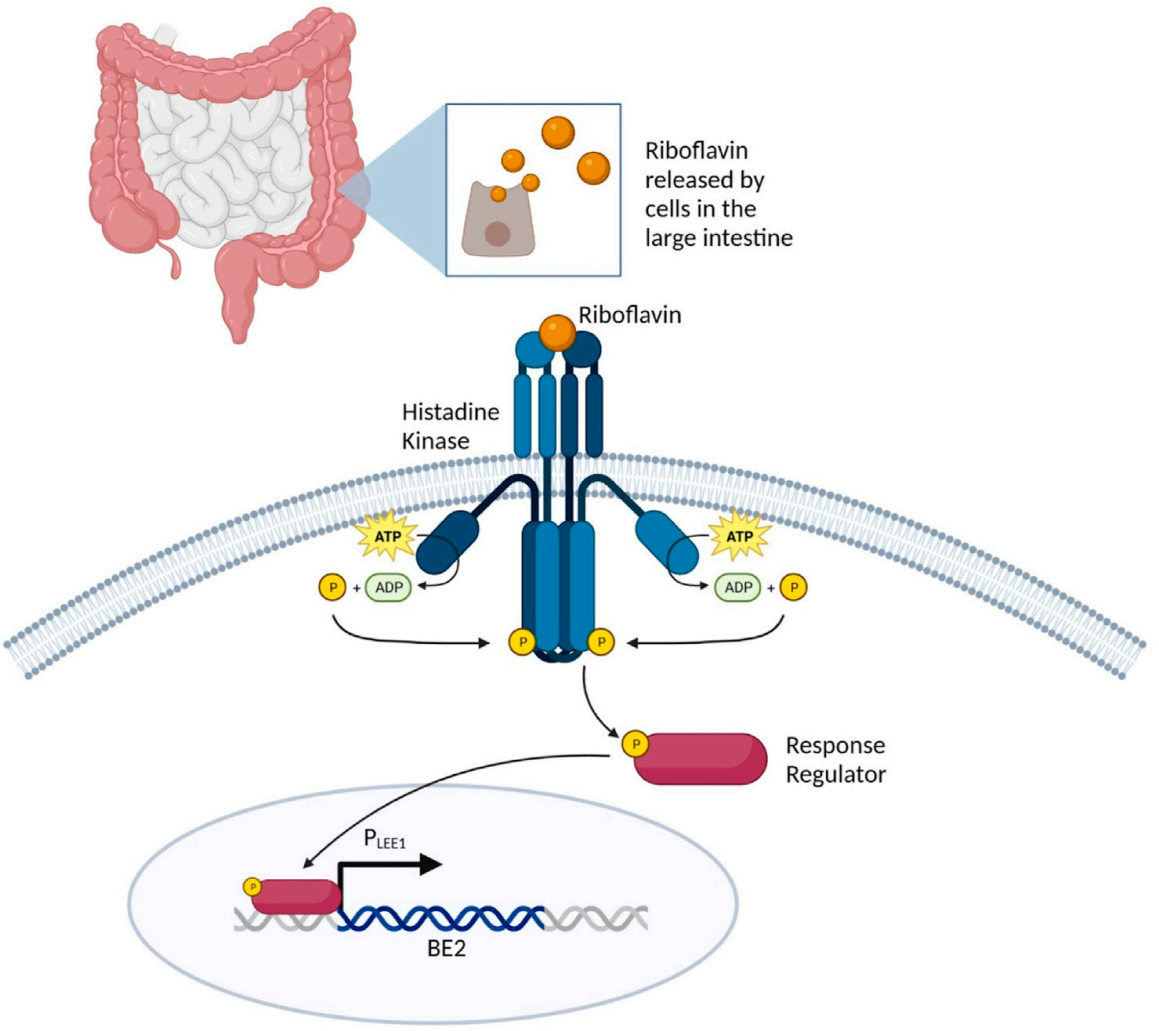
Overview of riboflavin-inducible sensing system to achieve large intestine specificity. Riboflavin in the large intestine induces HK, resulting in the autophosphorylation of the histidine residue in the RR protein. The phosphorylated RbfR undergoes a confirmation change that enables for specific binding to the LEE1 promoter.

The same study conducted by Liu et al. (2022) investigated how enterohemorrhagic *E. coli* O157:H7 acquired a two-component regulatory system (TCS) termed RbfSR, which induces the expression of LEE (locus of enterocyte effacement) genes through PLEE1 in the presence of riboflavin in the large intestine (Elliott et al., 2000; Platenkamp and Mellies, 2018; Liu et al., 2022). TCS is a basic stimulus-response coupling mechanism that allows many prokaryotic and some eukaryotic organisms to sense and respond to changes in various environmental conditions via phosphotransfer schemes. This phosphotransfer scheme involves histidine sensor kinase (HK), which detects the input, and a response regulator (RR) protein which produces an output. HK is impacted by a characteristic stimuli specific to the TCS; in the case of RbfSR TCS, it is induced by riboflavin (Stock et al., 2000). Once riboflavin binds to HK, the histidine residue is autophosphorylated, creating a high-energy phosphoryl group which is then transferred to an aspartate residue in the RR protein (Figure 4). This phosphorylation induces a conformational change in the regulatory domain that results in the specific binding of phosphorylated RbfR to PLEE1 (Liu et al., 2022).

This TCS does not share elements with the detection systems for the selected metabolites of interest, with the exception of butyrate, which can activate PLEE1 through the induction of pchA expression (Platenkamp and Mellies, 2018; Bai and Mansell, 2020). To prevent cross-talk between the butyrate and riboflavin systems, a mutant *E. Coli* strain that cannot express pchA will be used.

##### 2.2.2.3 Construction of the AND-Gate functionality by independent induction of Cas9 and sgRNA

The newly characterized TCS enables targeted sensing of metabolites in the large intestine due to the specificity of riboflavin and its independence from the promoter systems used to achieve sensing of the proposed metabolites of interest (Liu et al., 2022). To achieve this, we propose for the expression of BE2 to be induced by riboflavin-mediated activation of PLEE1, while sgRNA2 will be induced by the metabolite of interest (Tang and Liu, 2018). By linking the expression of each component to a separate promoter, AND-gate functionality can be achieved; base editing requires the activation of both promoters, which will only occur within the large intestine. Based on this design, the presence of any metabolites of interest in other gut regions will not initiate base editing, as BE2 will only be produced while the biosensor is within the large intestine.

It should be noted that this system design could feasibly allow for the simultaneous detection of multiple metabolites within a single bacterium by linking each metabolite to a separate sgRNA, and as such, a distinct base editing site (Tang and Liu, 2018). We have elected to restrict our design to include one metabolite per bacterium for two primary reasons: 1) it minimizes competition that would arise from having many different sgRNAs simultaneously compete for available BE2 (Tang and Liu, 2018), and 2) it ensures that there is sufficient space on the write plasmid to encode both the sensing and editing system, especially for large multi-component sensing systems. To quantify multiple metabolites, a new compartment containing a separate bacterial strain and its respective plasmid can be used.

##### 2.2.2.4 Directed base-editing normalizes for confounding due to riboflavin concentrations

Integrating individual sensors into an ingestible system requires information about metabolite concentrations to be encoded in a way that can be quantified independently from confounding factors such as variance in measurement duration and bacterial growth. Since recording is dependent on both BE2, which is regulated by riboflavin, and sgRNA, which is regulated by metabolite concentrations, the final base-editing ratio can be confounded by variations in riboflavin concentration. Given the situation where a metabolite of interest is assessed in an individual and found to be identical at two different time points, if riboflavin levels are not accounted for, the erroneous conclusion that metabolite levels have changed would be made. Hence, in order for meaningful concentration data to be extracted from base editing ratios, any variance in the induction of the riboflavin promoter relating to concentration must be corrected as a factor. To address this, an additional sgRNA, sgRNA1, will be linked to the riboflavin-sensing promoter (PLEE1) with a distinct editing target separate from sgRNA2, which is associated with metabolite sensing (Tang and Liu, 2018). This enables direct quantification of the amplitude of riboflavin exposure. Using this information, base editing ratios can be scaled in a way that reduces this effect.

#### 2.2.3 Kinetic model

The main goal of this model is to mathematically simulate the proposed base editing system to analyze its behavior in different environments, verifying that the system achieves AND-gate functionality, and thus, spatiotemporal specificity.

The model predicts how riboflavin and metabolite concentrations induce environment-specific plasmid editing in the large intestine. From the sensing of riboflavin and individual metabolites up to plasmid editing, the following major reactions were characterized in the model: riboflavin-regulated BE2 and sgRNA1 production, metabolite-regulated sgRNA2 production, sgRNA and BE2 dimerization, BE2 and recording plasmid complexation, and base editing (Supplementary Figure S1).

To simplify the simulation of the base editing system: 1) only one bacterium was included in the model, and 2) the sensing and recording of only one metabolite, indole, was simulated as a placeholder for other metabolites of interest.

##### 2.2.3.1 Methods

###### 2.2.3.1.1 Transcription

Mass action kinetics were used to model gene transcription. A steady state assumption was made, where the concentration of transcription reactants is not rate-limiting. This yields the following equation for the rate of mRNA production within one *E. coli* cell:

$$\frac{d\left[\text{mRNA}\right]}{\text{dt}}=k_{trsc}\times n_{RNAP}$$ (1)

where k_trsc_ represents the transcription rate in *E. coli*, and n_RNAP_ represents the number of RNA polymerases present in the cell.

For sgRNA and BE2 transcription, as they are positively regulated by their respective activators, the following form of the Hill equation was used:

$$\frac{d\left[\text{mRNA}\right]}{\text{dt}}=k_{trsc}\times n_{RNAP}\times \frac{{\left(\left[\text{Activator}\right]/K\right)}^{H}}{{1+\left(\left[\text{Activator}\right]/K\right)}^{H}}$$ (2)

where K represents the concentration of the activator that is required to achieve half-optimal activation power (i.e., the activation coefficient). H represents the Hill coefficient, which equals 1, since we assumed that the activator/DNA interaction exhibits no cooperativity.

###### 2.2.3.1.2 Translation

Applying mass action kinetics and the steady state assumption, gene translation was modeled with the following equation:

$$\frac{d\left[\text{Proteins}\right]}{\text{dt}}=k_{trsc}\times n_{Ribosomes}\times \left[\text{mRNA}\right]$$ (3)

where ktrsl represents the translation rate in *E. coli*, and nRibosomes represents the number of ribosomes present in the cell.

###### 2.2.3.1.3 Dimerization

Mass action kinetics were assumed for dimerization to yield a general equation representing the binding of the reactants and dissociation of the formed dimer:

$$\left.\frac{d\left[\text{Dimer}\right]}{\text{dt}}=k_{\mathrm{association}}\times [\text{Reactants}]\;-k_{\mathrm{dissociation}}\times [\text{Dimer}\right]]$$ (4)

kassociation and kdissociation are the association constant and dissociation constant, respectively.

###### 2.2.3.1.4 Degradation

A zero-rate assumption was made for the loss of mRNA (including sgRNA) and proteins; mRNA loss is dominated by decay, and protein loss is dominated by dilution. Mass action kinetics were used to yield:

$$\frac{d\left[\text{Species}\right]}{\text{dt}}={-k}_{\deg}\times \left[\text{Species}\right]$$ (5)

where k_deg_ is the degradation rate of the species in *E. coli*.

###### 2.2.3.1.5 Enzyme kinetics

The activity of CDA after formation of the BE2-plasmid complex was characterized as a constantly saturated enzyme: once sgRNA has directed the BE2-complex to bind to the plasmid, CDA is already positioned at the target base site, and as such, would not be subject to the stochastic interaction that normally precedes catalysis (Komor et al., 2016). Hence, the rate of plasmid editing, based on Michaelis-Menten kinetics, was modeled as:

$$\frac{\text{d}\left[\text{product}\right]}{\text{dt}}={\text{k}}_{cat}\times \left[Enzyme\right]$$ (6)

where kcat is the turnover number or catalytic rate constant. We assume that the behavior of this enzyme is not dependent on the specific sgRNA.

###### 2.2.3.1.6 Constants

Kinetic constants were sourced from the literature (Supplementary Table S1) (Bakshi et al., 2012; Bremer and Dennis, 2008; Hall et al., 2011; Krzysztoń et al., 2019; Liu et al., 2022; Matulis et al., 2022; Outten and O’Halloran, 2001; Raper et al., 2018; Tang and Liu, 2018; Yu et al., 2006).

###### 2.2.3.1.7 Final output

The final output of the model is the ratio of base editing at the indole and riboflavin locations, calculated based on the equation:

$$\text{ratio}=\frac{\left[\text{Edited}\;\text{Plasmids}\right]}{\left[\text{Edited}\;\text{Plasmids}\right]+\left[\text{Unedited}\;\text{Plasmids}\right]}$$ (7)

In the model, Ratio 1 is the base editing ratio of the riboflavin-associated recording site (mutation at position 166 on the eGFP gene), which is represented by Recording_Plasmid_1. Ratio 2 is the base editing ratio of the indole-associated recording site (mutation at position 186 on the eGFP gene), which is represented by Recording_Plasmid_2. It should be noted that the species Recording_Plasmids_1 and Recording_Plasmids_2 represent distinct base editing sites on the same group of recording plasmids, all of which are R1 from the CAMERA systems (Tang and Liu, 2018).

##### 2.2.3.2 Modelling results

**FIGURE 5:**
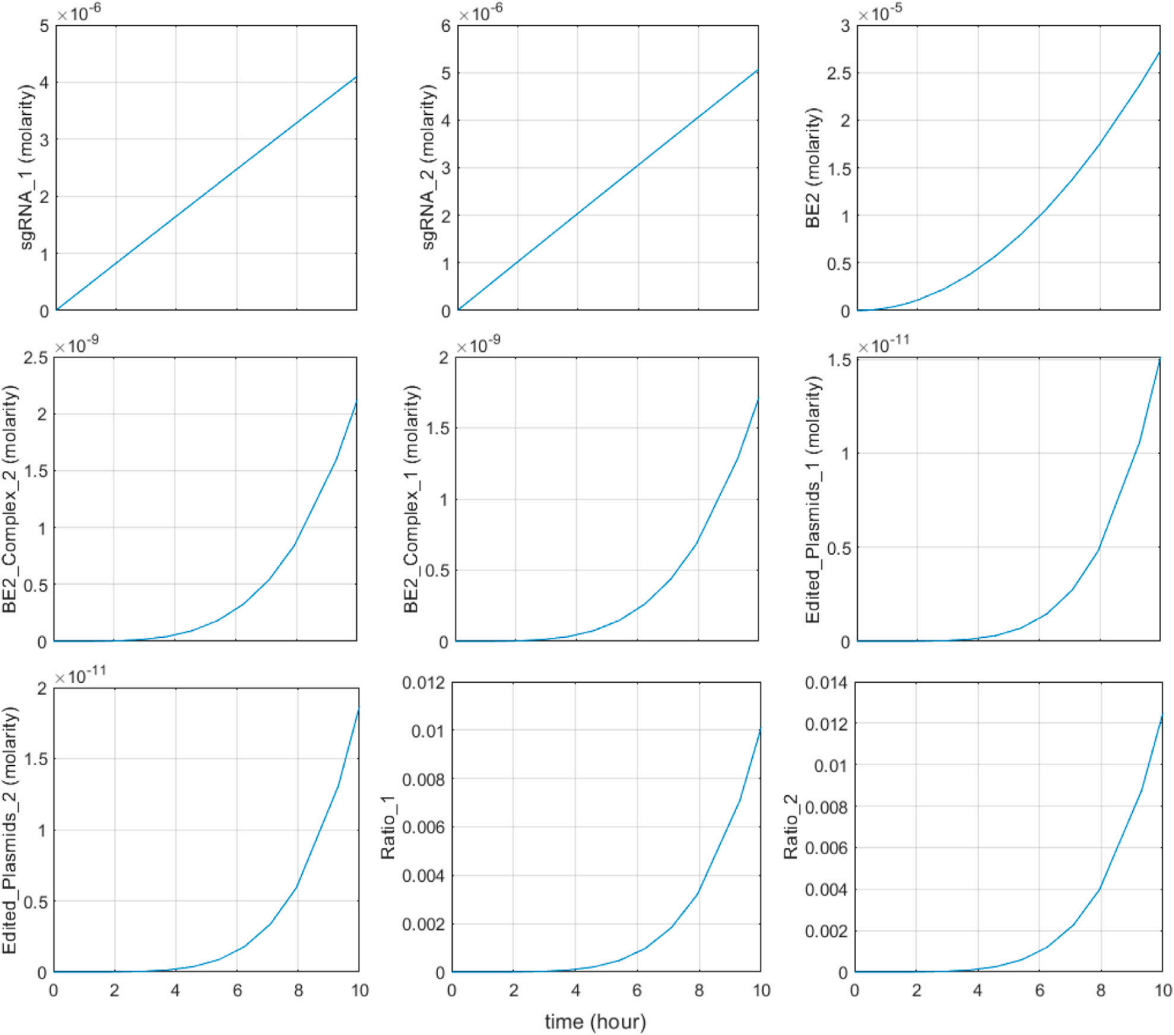
System response in typical large intestine conditions. Initial riboflavin concentration is 22.8 μM and initial indole concentration is 2.59 mM sgRNA1, BE2_Complex_1, and Edited_Plasmids_1 represent the riboflavin-associated species and Ratio_1 represents the base editing ratio of the riboflavin-associated recording site; sgRNA2, BE2_Complex_2, and Edited_Plasmids_2 are the indole associated species and Ratio_2 represents the base editing ratio of the indole-associated recording site.

**FIGURE 6:**
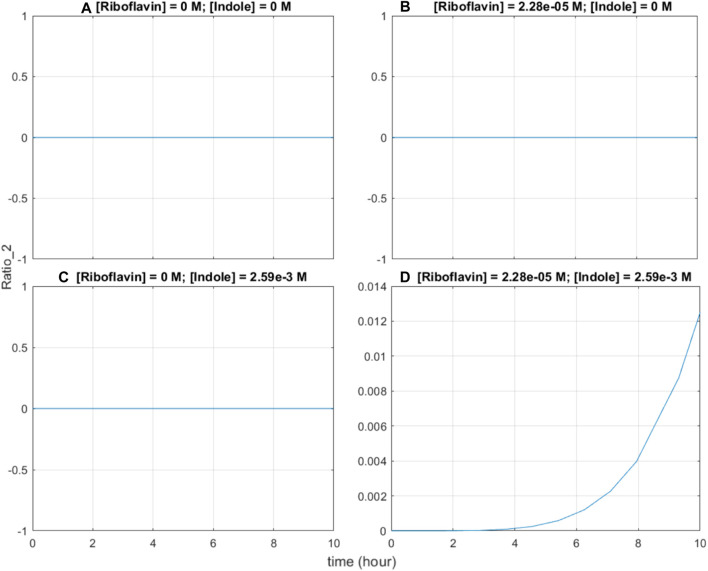
Base editing ratio of the indole-associated recording site under four test cases. **(A)** Both riboflavin and indole are absent. **(B)** Riboflavin is present at an initial concentration of 22.8 μM, while indole is absent. **(C)** Indole is present at an initial concentration of 2.59 mM, while riboflavin is absent. **(D)** Both riboflavin and indole are present in normal large-intestinal concentrations: initial riboflavin concentration is 22.8 μM, initial indole concentration is 2.59 mM.

**FIGURE 7:**
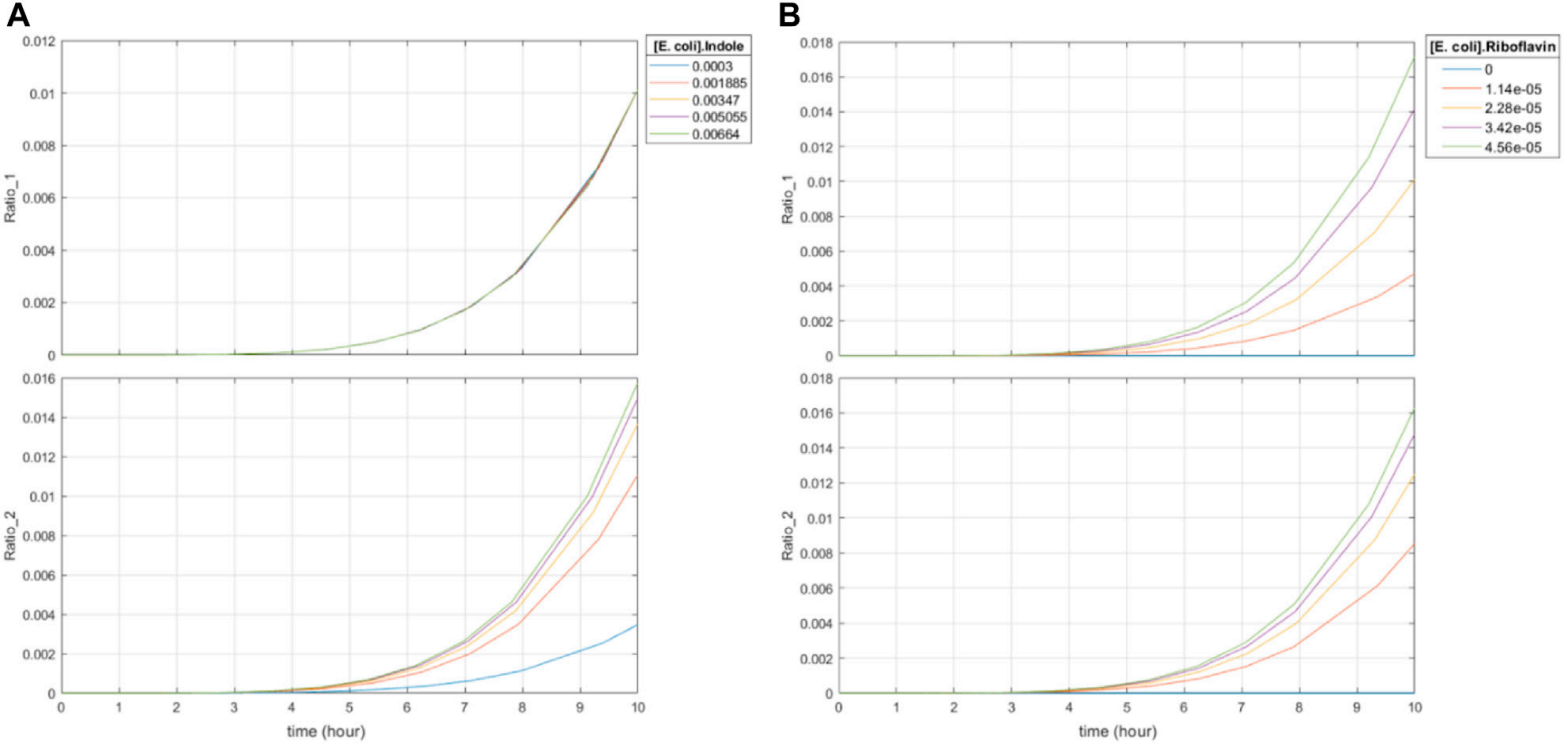
Effect of varying concentrations of riboflavin and indole on final base editing ratios. Ratio_1 is the base editing ratio of the riboflavin-associated recording site, and Ratio_2 is the base editing ratio of the indole-associated recording site. **(A)** 10-h simulation with constant initial concentration of riboflavin and varying initial concentrations of indole. Initial indole concentrations varied from 0.3 mM to 6.64 mM. **(B)** 10-h simulation with constant initial concentration of indole and varying initial concentrations of riboflavin. Initial indole concentrations were set to 2.59 mM. Initial riboflavin concentrations varied from 0 μM to 45.6 μM, which is the 0%–200% range of typical colonic riboflavin concentrations in mice, 22.8 μM (Liu et al., 2022).

**FIGURE 8:**
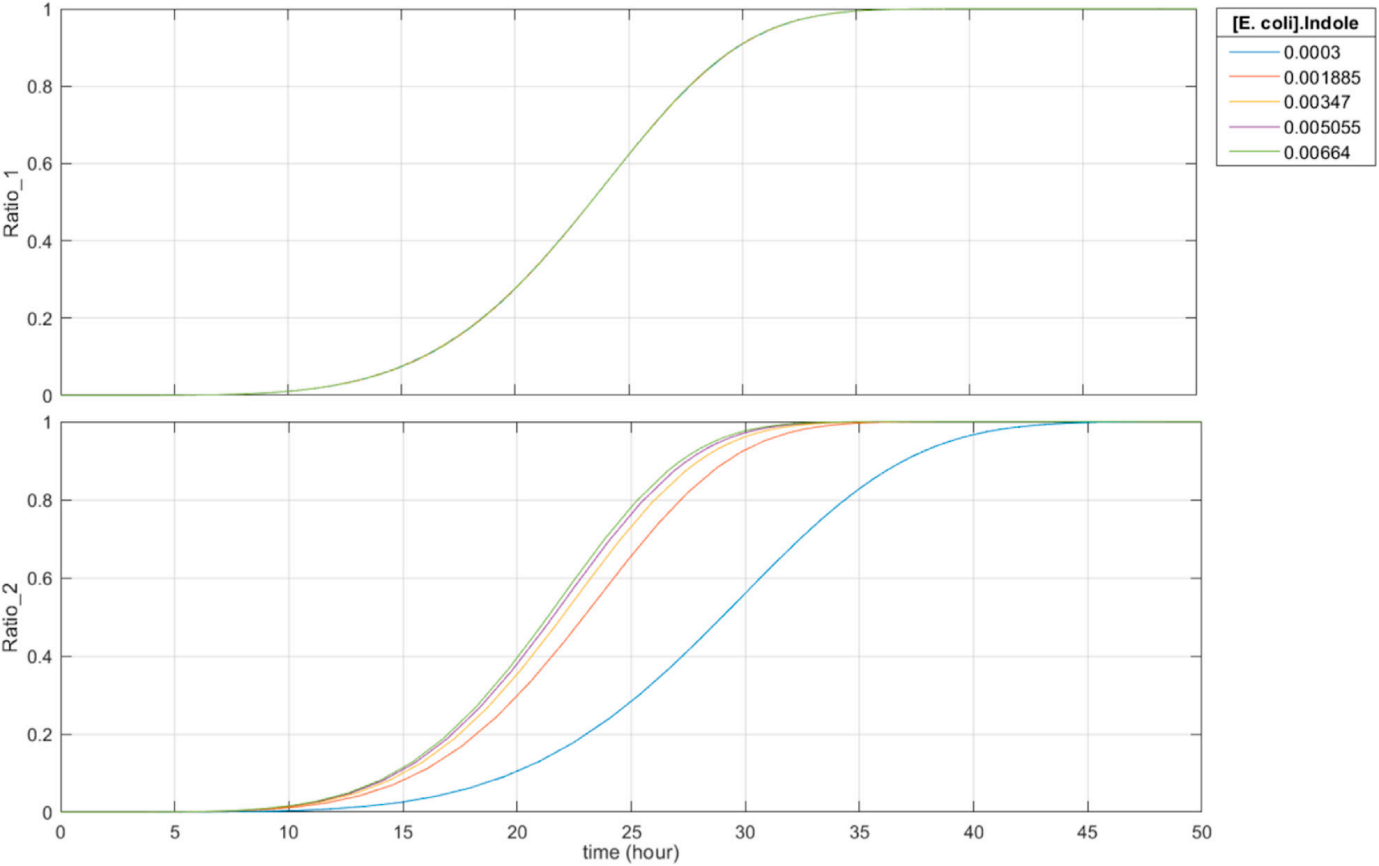
50-h simulation with constant initial concentration of riboflavin and varying initial concentrations of indole (0.30 mM–6.64 mM). Ratio_1 is the base editing ratio of the riboflavin-associated recording site, and Ratio_2 is the base editing ratio of the indole-associated recording site.

To validate the functionality of the base editing system, the model was tested with varying concentrations of the two inputs, riboflavin and indole, over a period of 10 h, similar to the expected time required for food to travel through the large intestine (Tobias and Sadiq, 2023). Initial concentrations of riboflavin were based on riboflavin concentrations in the colonic content of mice, 22.8 μM (Tobias and Sadiq, 2023). Indole concentrations were based on ranges found in human fecal samples: the mean fecal indole concentration in healthy individuals is 2.59 mM, though concentrations can vary widely, from 0.30 mM to 6.64 mM, among individuals (Darkoh et al., 2015). Concentrations of each metabolite exhibited negligible change during the 50-h simulation period. Results are shown in Figures 5–8, and demonstrate that the system behaves as expected in several key aspects, enabling targeted sensing within the large intestine.

First, the system responds to metabolite input as intended under typical large intestinal conditions. In Figure 5, the input metabolite concentrations were set to simulate normal large intestine conditions: initial indole concentrations were set as 2.59 mM, and initial riboflavin concentration was set to 22.8 μM. Expression of sgRNA1, sgRNA2, and BE2 occurs immediately in response to riboflavin and the metabolite, followed by the subsequent dimerization and complexation with recording plasmids, leading to increased concentrations of BE2-DNA complexes. The concentration of edited plasmids increases shortly after, confirming that base-editing occurs as a result of metabolite input.

Second, the base editing system exhibits AND-gate functionality; in theory, since base editing of the indole-associated recording site can only occur when both BE2 and sgRNA2 are expressed, it is expected that both riboflavin and indole are required to induce the system. In simulations where either riboflavin or indole are absent, and when both are absent, the graphs show no base editing activity (Figures 6A–C). Only when both riboflavin and indole are present, the base editing ratio of the indole-associated recording site increases (Figure 6D), verifying that the system functions as an AND gate.

Third, the system displays time- and dose-dependent editing ratios. Increased concentrations of indole with constant levels of riboflavin lead to increased base editing of the indole-associated recording site in a time- and dose-dependent manner (Figure 7A). Of note, the increases in base editing ratios were not linear with respect to input indole concentrations. Increased concentrations of riboflavin also lead to increased levels of editing at the riboflavin-associated recording site in a time- and dose-dependent manner (Figure 7B). However, it also causes increased editing at the indole-associated recording site on orders of magnitude similar to the editing changes due to changes in indole concentrations. This is congruent with the riboflavin-dependent expression of BE2, which exhibits first-order kinetics with respect to the final editing ratio by means of its dimerization with the indole-associated sgRNA. To this end, a normalization model is required to correct for this influence which will be discussed in the next section.

Fourth, recording of metabolite concentrations is robust past 15 h. As seen in Figure 8, when the initial concentration of riboflavin is constant and the initial indole concentration varies, the base editing ratios only start to become saturated at around 30 h. Since the expected time it takes for food to travel through the large intestine is 8–15 h, the recording duration of the system is suitable and robust for sensing within the large intestine.

Based on the results from the kinetic model, the system achieves AND-gate functionality, and base editing is dependent on metabolite concentration. It is expected that the base editing ratios fall within the order of 10^−3^ to 10^−2^ for both the riboflavin- and indole-associated recording sites. Overall, our results support the use of this system for recording metabolite concentrations in a location-dependent manner.

#### 2.2.4 A post-hoc normalization model corrects for riboflavin-concentration confounding and linearizes the relationship between actual and predicted metabolite concentration

**FIGURE 9:**
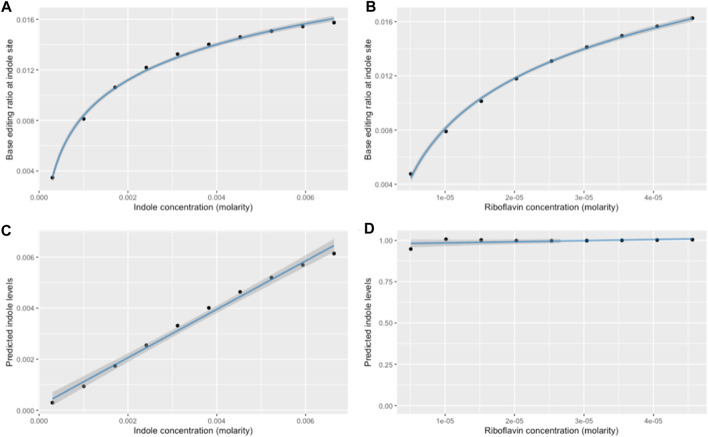
Results of normalization model for a 10-h simulation. **(A)** Logarithmic relationship between raw base-editing ratio at the indole site, and variations in indole concentrations from 0.3 mM to 6.64mM, and **(B)** variations in riboflavin concentrations from 0% to 200% of 22.8uM. **(C)** Comparison between actual indole concentrations and indole levels predicted after normalization. Riboflavin concentrations are constant, and units on the *y*-axis are arbitrary. **(D)** Predicted indole concentrations after normalization for various riboflavin concentrations. Actual indole concentrations are constant, and units on the *y*-axis are arbitrary.

Based on our kinetic simulation, the relationship between metabolite concentration and base-editing is logarithmic, not linear (Figure 9A). Furthermore, variations in riboflavin concentration would have a direct impact on base editing at the metabolite-associated recording site (Figure 9B). Thus, a normalization model was constructed to achieve two aims: 1) controlling the confounding effect of riboflavin concentration when using base-editing ratios to more accurately infer metabolite concentrations, and 2) creating a linear relationship between actual and predicted indole concentrations in order to more accurately assess fold-changes in metabolite concentrations. Data from the kinetic simulation at 10 h was used to determine the relationships and constants required for this model (Supplementary Table S2). This normalized model is intended to be deployed after extracting and reading the biosensor output; it uses final base editing ratios for each site as inputs, and returns a relative metabolite level with arbitrary units that has been normalized for riboflavin concentrations.

Based on exploratory analysis (Supplementary Figure S2), the relationship between riboflavin concentrations and base-editing at each of the two locations can be defined by:

$${\text{Ratio}}_{1}=\beta_{1\;}\cdot \sqrt{\left[\text{Riboflavin}\right]}+\alpha_{1}$$ (8)

$${\text{Ratio}}_{2}=\beta_{2\;}\cdot \;\log\left[\text{Riboflavin}\right]+\alpha_{2}$$ (9)

Hence, to nullify the relationship between riboflavin and ratio 2, both sides are divided by the right-hand side of Eq. 9:

$$\frac{{\text{Ratio}}_{2}}{\beta_{2\;}\cdot \log\left[\text{Riboflavin}\right]}=1$$ (10)

Substituting from Eq. 8:

$$\text{Normalized}\;\text{ratio}=\frac{{\text{Ratio}}_{2}}{\beta_{2\;}\cdot {\log\left[\frac{{\text{Ratio}}_{1}-\;{\alpha \;}_{1}\;}{\beta_{1\;}}\right]}^{2}+\alpha_{2}}$$ (11)

Given the logistic relationship between the normalized ratio and indole concentrations (Supplementary Figure S3), the prediction was linearized using:

$$Predicted\;=\exp\;\left(\frac{Normalized\;ratio-\beta_{3}}{\alpha_{3}}\right)$$ (12)

Linear regression showed that this model achieves a strong, linear relationship between the actual and predicted indole concentration when varying indole levels (Figure 9C: R^2^ = 0.992, *p* = 1.19 × 10^−9^). Furthermore, this model successfully removes the confounding effect of riboflavin: when indole was held constant and riboflavin was increased, a logarithmic increase in base-editing at the indole site (Figure 9B) was normalized such that a linear regression model demonstrated no significant relationship between riboflavin and predicted indole levels (Figure 9D: *p* = 0.149).

#### 2.2.5 Model considerations

##### 2.2.5.1 Limitations of the normalization model

This normalization model assumes that the duration for which recording takes place is the same between the two measurements. However, depending on various conditions, objects may take 8–15 h to travel through the large intestine (Tobias and Sadiq, 2023). Given that base editing is sensitive to both the duration and amplitude of a stimulus (Figures 7, 8), comparisons between base editing ratios would be invalid if the biosensor takes a significantly different amount of time to pass through the large intestine between two measurements; it is confounded by differences in recording duration. The base editing ratio of the riboflavin-associated recording site alone provides an insufficient baseline to control for both recording time and riboflavin concentration as the two confounders have unrelated effects on base editing: increasing riboflavin concentrations leads to a logarithmic increase in metabolite-associated base editing (Supplementary Figure S2B), while increasing time leads to an exponential increase (Figure 8). As such, the relative metabolite concentrations inferred from base editing through this current model will only be valid for comparisons between two measurements with the same recording duration.

##### 2.2.5.2 Limitations of the kinetic model

The kinetic model itself also has several limitations in addition to the assumptions pertaining to specific kinetic constants (Supplementary Table S1).

First, as we were unable to model base editing of one plasmid at both the indole- and riboflavin-associated recording sites, two separate species representing the same recording plasmids were created to clearly track base editing in response to each metabolite: Recording_Plasmids_1 represents base editing at the riboflavin-associated site, and Recording_Plasmids_2 represents base editing at the indole-associated site. It was assumed that each recording plasmid species could only be edited once.

Second, the model represents a single cell, and does not account for bacterial growth or decay: we assume that the findings from a single cell are generalizable to the behavior of individual cells in a bacterial population. Since the amount of metabolite sequestered by bacterial receptors is negligible compared to typical concentrations, bacterial growth should not affect the effective concentration of metabolites. However, changes in the physiology of the cell during growth have been shown to affect the performance of synthetic constructs, which could limit the applicability of the simulated results (Borkowski et al., 2016).

Third, the base editing ratios predicted by the model are limited by time. As noted earlier, the final base editing ratio after 10 h is typically on the order of 10^−2^, which is fairly low compared to the percentages achieved by the CAMERA systems (Tang and Liu, 2018). By increasing the run time of the model simulations, we found that the base editing ratios of our system increased exponentially. Furthermore, CAMERA 2.7 achieved 57% base editing for recording light exposure after a time period of 3 days (Tang and Liu, 2018). Since our proposed system functions in a substantially shorter time frame, it is reasonable to expect lower base editing ratios.

### 2.3 Proposed pill design

For the delivery of our ingestible biosensor, we propose a theoretical blueprint for a multi-compartment pill that will allow for the simultaneous sensing of multiple metabolites. The composition of the pill will be divided into two components: the outer coating and inner scaffolding. Additionally, the size of the pill is an important consideration as it influences both the volume for bacterial growth and comfort of ingestion for patients during clinical studies.

1. **Outer Coating**. This is needed to protect the contents from the harsh conditions of the GI tract. Here we propose the use of Eudragit^®^ L100, a pH-sensitive enteric coating, to protect the biosensor for more than 20 h in acidic artificial gastric fluid as described by De La Paz et al. (2022).
2. **Inner Scaffolding**. This allows for the transfer of metabolites but entrapment of bacterial sensing systems. We propose a multi-compartment scaffolding design within the pill composed of bacterial cellulose-based polymer nanocomposites which are porous but will not be degraded in the digestive tract (Kamel and Khattab, 2020). Bacterial cellulose is natively composed of fine pores up to 0.02 µm depending on preparation methods (Revin et al., 2022). As most *E. coli* are 1.0–2.0 µm long with radius of about 0.5 µm, the polymer can be tailored to exclude *E. coli* translocation between membranes while enabling the passive diffusion of smaller compounds such as glucose (0.001 µm) and gut metabolite biomarkers (National Research Council, 1999). Specifically, type-I cellulose exhibits high crystallinity, thermodynamic stability, and tensile strength with a Young modulus of 15–18 GPa. This allows the pill to overcome the high stress-inducing environment of the GI tract due to peristalsis (Gorgieva and Trček, 2019; Li et al., 2019). The use of bacterial cellulose also prioritizes safety, due to its biocompatible, biodegradable, and non-toxic nature.
3. **Size of the pill**. This variable can influence both the patient experience as well as plasmid quantity after extraction. A size of 11 mm × 26 mm has been described in previous studies, which allows for a total volume of 2.122 mL within the pill (Van Gossum et al., 2009; Fields et al., 2015; De La Paz et al., 2022). As described later, we propose an MDD-tailored, five metabolite panel; this would allow for a volume upwards of 0.424 mL per compartment which is adequate for bacterial growth provided proper nutrition is available.

### 2.4 Extraction of recorded data for quantification

In order to analyze the stored data within the recording plasmids and make meaningful conclusions about gut metabolite concentrations, we propose the following protocol. After collection of the pill, each compartment will be individually grown in separate liquid media, allowing for further growth of the sensing system. As mutations on recording plasmids do not influence the reproductive fitness of the *E. coli* sensing system, bacteria with different proportions of edited to unedited recording plasmids should grow at equal rates (Tang and Liu, 2018). Standard protocol of plasmid extraction will be conducted by researchers where the eGFP gene will be specifically amplified using polymerase chain reaction (PCR) techniques. The resulting amplicon will be analyzed using high throughput sensing with a sequencing depth of 50,000 copies. For each of these amplicons, 300 bps will be sequenced (150 bp on each end). Sequence variation amongst the copies will allow for researchers to calculate the proportion of mutated to unmutated nucleotides of specific sites within the eGFP fragment. Finally, the resulting portions can be used to back calculate the initial concentrations of the metabolite of interest as well as the concentration of riboflavin required to normalize the resulting data.

### 2.5 Proposed MDD associated metabolite disease panel

In this section, we outline five specific gut metabolites that have the potential to direct our proposed biosensor for MDD; incorporating their respective sensing systems will effectively create an ingestible *in vivo* assay for MDD.

#### 2.5.1 Butyrate concentrations in the gut as a predictor of MDD

The short chain fatty acid (SCFA), butyrate, is a gut-derived metabolite that has shown promising potential in alleviating depressive symptoms in patients with MDD through its anti-inflammatory properties. Additionally, studies have shown that butyrate-treated microglia respond to LPS stimulation by decreasing the production of cytokines such as TNFa and overall dampening neural inflammation (Park et al., 2019; Silva et al., 2020; Caetano-Silva et al., 2023).

Studies also point to the role of butyrate in the stimulation of afferent fibers of the vagus nerve, which has been shown to suppress depressive symptoms in mild and severe MDD patients (Peña et al., 2014; Stilling et al., 2016; Bonaz et al., 2017; 2018; Cawthon and De La Serre, 2018). Though the mechanism of action is not fully elucidated, some hypothesize that this stimulation alters the expression of receptors such as GPR41, which is found on enteric nerve fibers and interacts with SCFAs to allow for direct communication from the gut to the CNS (Schroeder and Bäckhed, 2016; Bruning et al., 2020).

Studies have shown that butyrate-producing Faecalibacterium and Coprococcus were consistently found at lower levels in MDD patients compared to controls (Jiang et al., 2015; Zheng et al., 2016; Valles-Colomer et al., 2019). Specifically, F. prausnitzii, a butyrate producing bacteria that comprises up to 14% of the total fecal gut microbiota (Rivière et al., 2016), was found at relatively decreased levels across multiple studies (Jiang et al., 2015; Zheng et al., 2016; 2020; Chen et al., 2018; 2020; Rong et al., 2019; Stevens et al., 2021). This was further supported by a study with 165 young adults, which showed a negative association of fecal butyrate concentrations with depressive symptoms (r = −0.195) (Müller et al., 2021).

##### 2.5.1.1 Butyrate sensing system

**FIGURE 10:**
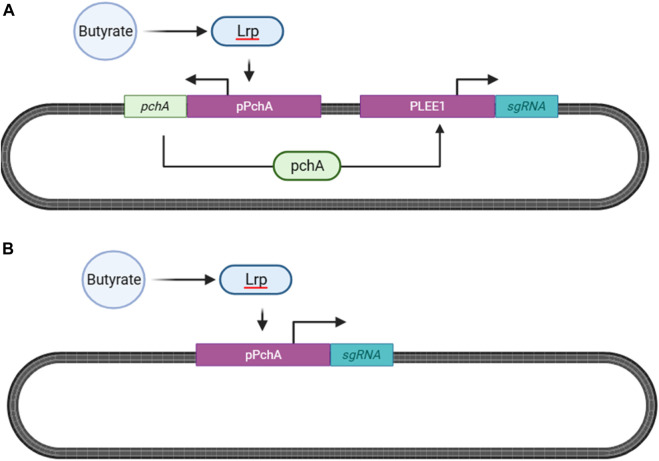
Butyrate Sensing in *Escherichia coli* Nissle 1917 (EcN, serotype O6:K5:H1). **(A)** Butyrate and the leucine-responsive regulatory protein (Lrp) form a binary complex which binds to the promoter region of PchA (pPchA) (Henker et al., 2008; Gaudier et al., 2009). In the system by Bai and Mansell, 2020, PchA subsequently binds to the LEE1 promoter that then natively initiates the transcription of the gene of interest (Bai and Mansell, 2020). **(B)** Butyrate sensing system adopted from Serebrinsky-Duek et al., 2023, to avoid coactivation of the riboflavin system, with the reporter gene (sgRNA) directly downstreamof pPchA (Serebrinsky-Duek et al., 2023).

The system designed by Bai and Mansell (2020) is the most established butyrate sensing system in literature, exhibiting high sensitivity for butyrate over other SCFAs. In this bidirectional system, butyrate first forms a binary complex with the leucine-responsive regulatory protein (Lrp), which then binds to the promoter region of pchA (PpchA) resulting in PchA transcription. PchA subsequently binds to PLEE1 resulting in the transcription of the gene of interest (Figure 10A).

However, as our previously discussed riboflavin sensor is also dependent on PLEE1 to transcribe the ler gene, crosstalk between the two sensing systems can result in confounding output. As such, we propose implementing the sensing system created by Serebrinsky-Duek et al. (2023), which places the reporter gene downstream of PpchA (Figure 10B). According to the authors, upon deletion of pchA, butyrate-mediated induction of PLEE1 did not occur, mitigating potential crosstalk between the individual sensing systems.

One limitation of this system is that the deletion of pchA results in a leaky PpchA promoter that causes high basal transcription rates in the absence of butyrate (Serebrinsky-Duek et al., 2023). Therefore, during *post hoc* analysis, butyrate base-editing ratios should be normalized by basal PpchA activity for accurate interpretation of results. Additionally, the ability of this system to distinguish butyrate concentrations with high specificity is limited; therefore, optimization of this sensing system via site directed mutagenesis is required before implementation.

#### 2.5.2 Tryptophan pathway metabolites

Tryptophan, an essential amino acid necessary for protein synthesis, plays a key role in the GBA; specifically, its metabolic pathway produces three critical metabolites: kynurenine, indole, and serotonin (Roth et al., 2021). The majority of tryptophan (95%) undergoes conversion into kynurenine which is subsequently degraded into neuroprotective kynurenic acid and neurotoxic quinolinic acid (Badawy, 2017; Savitz, 2019), whereas serotonin and indole metabolites are produced to a lesser extent. The intricate relationship between the tryptophan pathway and MDD is demonstrated by a breadth of research.

##### 2.5.2.1 Serotonin

The link between serotonin (5-HT) and MDD was initially supported by studies on serotonin depletion in cerebrospinal fluid (CSF) (Cheetham et al., 1991) and reduced serotonin uptake in depressed patients (Healy and Leonard, 1987). However, a collection of recent meta-analyses evaluated by Moncrieff et al. (2022) via sensitivity analysis, revealed no significant distinction in 5-HT levels between individuals with and without depression, challenging the link between 5-HT and MDD. Other studies also have found no statistical significance between MDD and healthy controls to serotonin metabolites such as 5-hydroxyindoleacetic acid in the CSF (Ogawa et al., 2018; Pech et al., 2018). Thus, due to conflicting findings on serotonin-MDD association and sensing system development difficulties, we find that it is more advantageous to pursue alternative metabolites in the tryptophan pathway (Lengger et al., 2022). Yet, after optimization of our biosensor, further clinical *in vivo* studies can be conducted to better elucidate the relationship between serotonin and MDD.

##### 2.5.2.2 Indole concentrations in the gut as a predictor of MDD

Indole, an intercellular signaling molecule fundamental to the gut microbiome, facilitates a host of functions including drug resistance, plasmid stability, biofilm formation, and virulence control in pathogenic bacteria (Lee and Lee, 2010). Indole enhances IEB barrier integrity by augmenting the expression of genes for tight junctions, the actin cytoskeleton, and adherens junction (Bansal et al., 2010; Shimada et al., 2013). Additionally, indole plays a role in the balance of both proinflammatory anti-inflammatory effects (Al-Sadi and Ma, 2007; Bansal et al., 2010).

The overproduction of indole by gut microbiota was observed to induce consistent anxiety-like behavior and mood disorders in rats and mice (Jaglin et al., 2018; Mir et al., 2020). In addition, indole was found to induce a dramatic increase of the expression of the adrenomedullary Pnmt gene, which is involved in catecholamine biosynthesis, exhibiting an increased vulnerability of male mice to the adverse effects of chronic mild stress on emotional behaviors (Mir et al., 2020). Disruption of the balance of the intestinal serotonergic system can also contribute to gut inflammation. Alistipes, an indole-positive species that limits serotonin production, was found in abundance in MDD patients and proposed to be associated with gut inflammation (Jiang et al., 2015). In a study by Delgado et al. (2022), lower levels of the indole derivative indole-3-carboxaldehyde, was correlated with increased severity of depressive symptoms in obese patients with depression.

###### 2.5.2.2.1 Indole sensing system

**FIGURE 11:**
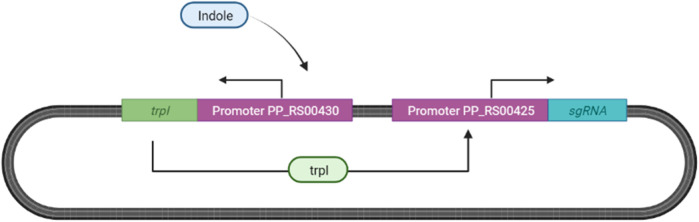
Indole Sensing in *Escherichia coli*. Indole binds to PP_RS00430, inducing the expression of transcription factor (TrpI) allowing for the expression of tryptophan synthase subunits, now substituted with sgRNA located after the promoter PP_RS00425 (Matulis et al., 2022).

We propose adopting the indole responsive gene expression system designed by Matulis et al. (2022) for our ingestible biosensor as described in Figure 11.

##### 2.5.2.3 Hydrogen peroxide concentrations in the gut as a predictor of MDD

The kynurenine pathway, which is initiated by the rate-limiting enzyme indoleamine 2,3-dioxygenase (IDO), produces downstream metabolites including kynurenic acid and quinolinic acid, however, these metabolites are not found in measurable quantities in the gut lumen (Sublette et al., 2011; Valladares et al., 2013; Johnson et al., 2021). IDO on the other hand is expressed in the small intestine, and is known to have modulatory effects on neurotransmission at elevated levels; in fact, IDO inhibition results in decreased production of kynurenine and the suppression of proinflammatory cytokines (Valladares et al., 2013).

A study by Johnson et al. (2021) explored the amelioration of MDD symptoms correlated with IDO inhibition, decreased kynurenine, and increased *Lactobacillus*, which occurs through the mechanism of H2O2 secretion. *Lactobacillus* is known to have a beneficial effect on depressive disorders, and is negatively correlated with hypothalamic-pituitary-adrenal (HPA) axis response and hyperactivity. Valladares et al. (2013) further confirms these findings in a study that monitored changes in IDO concentrations in intestinal epithelial tissue of mice exposed to L. johnsonii which excretes H2O2 as a byproduct of its pyruvate oxidase function. Additionally, the bacterium catalyzes pyruvate into the form of acetyl phosphate, which produces the byproducts CO2 and H2O2 (Cornacchione and Hu, 2020). The results showed a 47% reduction in IDO activity, with a 3.9 fold increase in ileum lumen H2O2 (Valladares et al., 2013). H2O2 activates the heme peroxidase function of IDO, consequently deactivating its dioxygenase function, explaining the reduction in IDO activity (Freewan et al., 2013). This evidence suggests a link between H2O2 production and IDO inhibition, which may play a role in the exacerbation of MDD (Bajpai, 2014).

###### 2.5.2.3.1 OxyR sensing system

**FIGURE 12:**
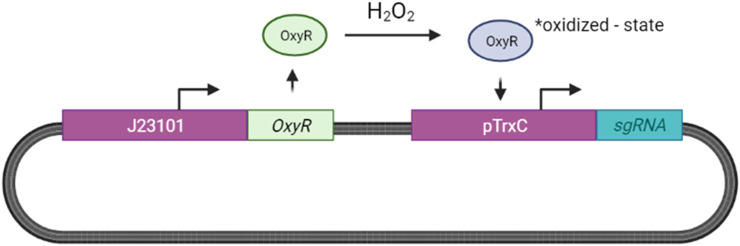
H2O2 Sensing in E. coli. Increased H2O2 levels result in the activation of the OxyR protein, resulting in the formation of a disulfide bond between two OxyR cysteine residues (Matulis et al., 2022). This activated OxyR state results in the activation of the TrxC promoter (pTrxC), leading to sgRNA expression.

We propose adopting the OxyR sensing system for H2O2 designed by Wei et al. (2012) for our ingestible biosensor as described in Figure 12.

#### 2.5.3 Tetrahydrofolate concentrations in the gut as a predictor for MDD

Tetrahydrofolate (THF), a folate (vitamin B9) derivative and precursor to L-methylfolate (5-MTHF), is a metabolite produced by gut microbiota for various essential biosynthetic processes such as DNA and amino acid synthesis (Khan and Jialal, 2022). Neural cells also utilize 5-MTHF, taken up through folate receptors and transporters, for crucial processes such as neural cell division, and tissue repair (Balashova et al., 2018). Additionally, 5-MTHF binding triggers specific signaling mechanisms such as neurulation (Ami et al., 2016), and modulates synaptic activity. THF deficiency can therefore lead to impairment of cell division and neurological disorders (Khan and Jialal, 2022).

Approximately one-third of depressed individuals are folate deficient (Young and Ghadirian, 1989; Bottiglieri et al., 1992; Bottiglieri et al., 2000). Presently, studies support the link and suggest that folate supplementation, particularly with 5-MTHF, is beneficial for depressed patients (Nelson, 2012; Bender et al., 2017; Altaf et al., 2021). Furthermore, a mutation in the MTHFR gene, an enzyme required to convert 5-MTHF from THF, was found in MDD patients leading to 5-MTHF unavailability (Gilbody et al., 2007a; Gilbody et al., 2007b). Additionally, 5-MTHF adjunct therapy with selective serotonin reuptake inhibitors (SSRIs) or serotonin-norepinephrine reuptake inhibitors (SNRIs) was investigated and proven effective in MDD patients (Altaf et al., 2021; Lam et al., 2022), increasing remission rates by 39% compared to patients who only received SSRIs or SNRIs.

It is believed that 5-MTHF supplementation benefits MDD patients through tetrahydrobiopterin (BH4) regeneration (Altaf et al., 2021) as 5-MTHF is further processed into BH4 in the brain (Akoglu et al., 2008; Miller, 2008). BH4, a rate-limiting cofactor for hydrolase enzymes such as tyrosine and tryptophan hydrolase, is required for the synthesis of various monoamine neurotransmitters including serotonin, dopamine, norepinephrine, and epinephrine (Miller, 2008). Lower BH4 levels are also found in depressed individuals (Coppen et al., 1989; Anderson et al., 1992; Nasser et al., 2014), further implying a relationship between BH4 and MDD.

##### 2.5.3.1 THF sensing system

**FIGURE 13:**
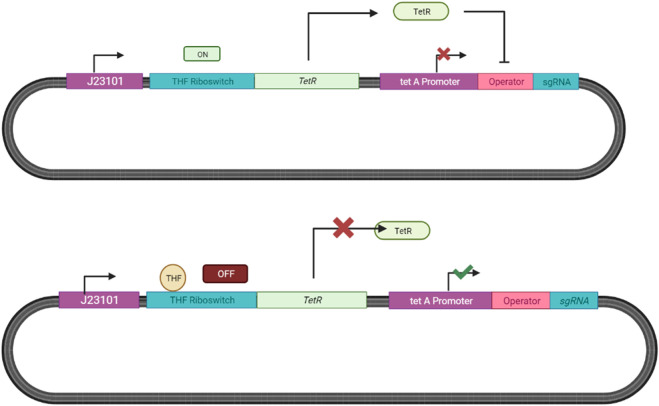
Tetrahydrofolate Responsive Riboswitch System in E. coli. Endogenous E. coli RNA polymerase binds to the constitutive J23101 promoter, transcribing the THF riboswitch. THF present then binds to the riboswitch terminating the expression of the tet repressor (TetR), leading to the transcription of sgRNA after the promoter tetA (Leigh, 2015; Das et al., 2016). In the absence of THF the repressor is expressed and inhibits sgRNA expression.

We propose adopting the THF responsive riboswitch system designed by Leigh (2015) paired with the Tet-On inducible system by Das et al. (2016) for our ingestible biosensor as described in Figure 13.

#### 2.5.4 Tetrathionate concentrations in the gut as a predictor of MDD

Finally, we propose tetrathionate as a novel indicator for MDD. Tetrathionate is a commonly used marker of gut inflammation for diseases such as inflammatory bowel disease (IBD). There exists a fair library of research linking MDD to IBD and its subtypes, Crohn’s disease (CD) and ulcerative colitis (UC). Shared factors between the two disorders include the dysregulation of the GBA (Fadgyas-Stanculete et al., 2014), microbiota and IEB function (Jeffery et al., 2012; Kelly et al., 2015; Yuan et al., 2021), stress (Mawdsley and Rampton, 2005; Goodhand et al., 2012), immune dysregulation (Persoons et al., 2005), and increased circulating proinflammatory cytokines (Alexakis et al., 2017; Facanali et al., 2023). Additionally, IBD and MDD also share disease implicated metabolites such as SCFAs and those involved in the tryptophan pathway (Banfi et al., 2021). Therefore, the relationship between tetrathionate and IBD can rationally be extended to MDD.

Studies show higher rates of depression in patients diagnosed with IBD compared to healthy controls (Goodhand et al., 2012; Byrne et al., 2017). Specifically, one study by Addolorato et al. (1997) showed a significantly higher percentage of individuals with depression in CD and UC patients than in controls (41.9% versus 11.1%, and 50.0% versus 11.1%, respectively). Some studies also indicate that disease activity is influenced by depression and that disease duration can be inversely correlated with MDD presence (Goodhand et al., 2012; Byrne et al., 2017; Facanali et al., 2023). Further, a prospective study by Persoons et al. (2005) found that after treatment with infliximab, an anti-TNF therapy, patients with MDD at baseline achieved remission significantly less (29%) in comparison to non-depressed patients (70%) and required re-treatment.

The pathology of IBD has eluded researchers for years, however, recently the possibility of MDD as a causal or resultant factor of inflammatory gut disorders has been introduced. For example, UC may potentially be caused by a disturbed inflammatory response toward a set of specific gut microbiota, or, gut microbiota may interact with the CNS through the immune system and circulating metabolites such as neurotransmitters can subsequently affect an individual’s mood (Yuan et al., 2021). Thus, there exists a space to explore the bidirectionality of the GBA and the many gut-brain or brain-gut mechanisms that may be in play (Collins, 2020; Günther et al., 2021).

##### 2.5.4.1 Tetrathionate Sensing system

**FIGURE 14:**
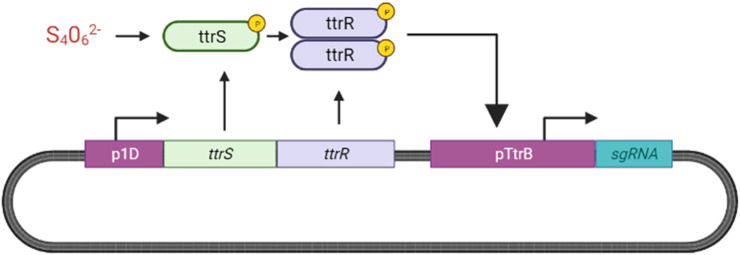
Overview of Tetrathionate Sensor. In the presence of tetrathionate, TtrS is phosphorylated, allowing for the phosphorylation and dimerization of ttrR. ttrR binds to the ttrB promoter in the tetrathionate reductase operon (ttrBCA) to activate transcription of downstream genes which we have replaced with sgRNA (Hensel et al., 1999; Daeffler et al., 2017).

We propose adopting the TtrSR sensing system designed by Daeffler et al. (2017) and Hensel et al. (1999) for our ingestible biosensor as described in Figure 14.

## 3 Discussion

MDD is a serious mental illness that affects a significant proportion of the global population. As such, the market for MDD treatments is a multifaceted one that must account for a variety of factors such as disorder prevalence, diagnosis accuracy, efficacy of current treatments, and the potential for new therapies. Therefore, it remains an untapped area with significant potential for growth and improvement in diagnostic aids.

In this work, we propose a design model for an ingestible bacterial system with temporal and spatial specificity for the large intestine, equipped with genetic-based reporter systems for long-term memory of acquired signals. In particular, the fully biological, CRISPR based, recording and writing systems form the core of the biosensor, while the other design functions can, in theory, be modified according to the user’s need. This creates a multiplex and modular system with several advantages, as listed below:

1. **Scalability of metabolites**. The number of target metabolites can easily be adapted by creating additional bacterial compartments to house each desired sensing system. In this way, the biosensor can easily be scaled up and eventually form disease specific panels.
2. **Metabolite selection beyond MDD**. By assigning each stimulus to a separate sgRNA, this biosensor can be designed to accommodate for any metabolite of interest that has a programmable sensing system. The use of AND/OR gates can also be employed in order to create further specificity and determine relationships between acting metabolites. Thus, this effectively creates a versatile ingestible *in vivo* assay for any disorder of interest that is associated with gut microbiota dysfunction.
3. **Spatiotemporality**. In this paper, spatial specificity is obtained via riboflavin sensing, as many of our proposed MDD-metabolites are found in the large intestine; however, our system can be tuned to include pH, O2, or other spatio-specific sensors to study local metabolite dynamics in a location dependent matter through any segment of the GI tract.
4. **Prospective clinical advancement**. Numerous mood disorders have been linked to the GBA, however, there is a severe lack of research that points towards pivotal players in the gut microbiome. A versatile biosensor, as proposed herein, holds promise to fill in these gaps in knowledge, serving as a vital tool for novel metabolite characterization. Thus, though this biosensor is currently geared towards researchers who are working towards bridging these gaps in the GBA, upon further evaluation and testing, there lies an immense potential for the clinical use of an ingestible biosensor to ultimately aid with accurate diagnosis of mood disorders such as MDD and subsequent personalized medicine.

### 3.1 Considerations for implementation

It is important to note that the proposed ingestible biosensor is in its developmental infancy, and more research and optimization is required before it becomes technologically mature. The internal DNA writing and CRISPR based editing system for the recording of metabolites has been validated in previous work along with the chosen sensing systems for gut metabolites, while the proposed interaction between the two systems along with specificity for a spatio-specific molecule is novel and was tested in this work using kinetic modeling. However, overall use of the tool for gut metabolite quantification has yet to be constructed and tested in the lab. Thus, though the components of our system are at different stages of development, our current work to date is likely at a Technology Readiness Level (TRL) of 1 (Government of Canada, 2021). Further experimentation to validate how the system will interact with the physical world and publishing of this data will be required before moving to the next stage.

Prior to implementation, regulatory and safety precautions within North America would require the completion of a new drug application approval as specified under section 3.2 (e) (1) of Title 21 of the American Code of Federal Regulations (Center for Drug Evaluation and Research, 2022). This process would ensure that the pill is safe for ingestion, and that the research benefits of the pill and its acquired data outweigh any potential complications (Center for Drug Evaluation and Research, 2022).

#### 3.1.1 Safety concerns surrounding the use of ingestible biosensors

One of the primary concerns regarding the use of ingestible biosensors lies in the logistics of device encapsulation and device retention within the GI tract. A majority of ingestible biosensors are composed of rigid and non-degradable materials, which can potentially result in damage to the GI tract should the device be retained for more than six to eight hours depending on individual GI movement (Watson, 2020; Lu et al., 2023). Approximately 1.4% of patients experience capsule retention, therefore, precautions such as utilizing soluble and malleable materials can be exercised to reduce rates of retention and blockage (Lu et al., 2023). This is important to consider as trauma to the gut can cause gastrointestinal perforations, leading to internal bleeding or peritonitis (Revell et al., 2018). However, as described earlier, our biosensor pill will be administered in a highly controlled clinical setting with stringent subject inclusion criteria. This will include screening out individuals with digestive or GI motility issues which can be monitored using barium X-rays as described previously (Palmer et al., 2011). If the pill is retained in the GI tract, it can be removed by either medications or endoscopic retrieval to prevent any potential complications associated with long-term capsule retention (Rondonotti et al., 2010).

The implementation of a killswitch has yet to be added to our system; this is important to consider as the system is biological instead of chemical or electrical. While the issue of patient privacy is avoided without the use of electronics, the concern of potential contamination either to the environment or the human body is of concern and must be addressed. Thus, future studies should consider implementing a system that can neutralize the bacteria via expression of toxins or lysis protein within the pill in the presence of an external chemical stimulus, as proposed by Rottinghaus et al. (2022). This ensures that in the given scenarios where a patient desires to withdraw from the study, the engineered bacteria can be safely disengaged.

It is important to note that this system as a whole remains an unvalidated hypothesis, therefore initial tests should focus on validating its functionality in a tightly-controlled *in vitro* environment. Furthermore, our design is also conducive for use in animal models to further test for functionality and safety (Turner et al., 2011). Specifically, large animal models are more appropriate than small rodent models, as the pill would not need to be dramatically resized and risk impacting its capacity and functionality. Additionally, porcine or canine models have demonstrated a more analogous GI tract to humans than mice (Ziegler et al., 2016; Ribitsch et al., 2020).

#### 3.1.2 Other considerations for applicability

There are several key factors that must be considered before any practical application is possible.

First, the ability to distinguish biologically relevant differences from non-ideal behavior of the bacteria (i.e., signal-to-noise ratio) is a large concern. Thus far, genetic reporter systems have been validated *in vitro* using high inducer concentrations to elicit strong promoter responses. When seeking to translate this technology for *in vivo* applications, a number of environmental effects will become relevant and influence the behavior and efficiency of the system. Differences in metabolites and salts in the bacterial environment, for example, may affect the ability of the Cas9 complex to associate with DNA (Zhang, S., et al., 2020). Cell-specific machinery may also exhibit inhibitory effects that can further modulate base-editing (Wang et al., 2017). At the same time, inducer concentrations will vary less drastically between groups, especially when investigating mood-disorder related biomarkers which often have small effect sizes that render many results non-significant (Kennis et al., 2020). Hence, the effect of these variations in comparison to the effect size of metabolites being studied must be considered to determine the degree of precision that is required to produce the desired response. It is also important to note that any quantitative data from our sensor that is collected is not useful in and of itself. The collected data must be correlated to MDD symptomatology and severity by monitoring individuals over time to provide useful insights applicable to studying the disease.

Second, the growth rate of the engineered sensing bacteria within each compartment must be considered as base editing is strongly dependent on the bacterial generation of plasmids containing each sensing system. There are a number of factors that can influence bacterial growth, of which the growth medium is the most pertinent. Based on past ingestible biosensors which have utilized a semi-permeable membrane to separate the bacterial compartment from the external environment (Mimee et al., 2018), nutrient availability will greatly depend on local GI environments; this can influence bacterial growth rate and therefore the generation of sensing plasmids. Additionally, the duration of time between ingestion to excretion, and within each local GI environment, can vary from individual to individual depending on a number of factors. As such, further studies using *ex vivo* and *in silico* models to simulate the gut microbiome and device trajectory through specific GI segments can be used to correlate ingestion duration with optimal bacteria growth phases. This can also be used to provide crucial information pertaining to the initial seeding density of bacteria within each compartment and determine if an optimal concentration is required prior to entering the target environment for optimal metabolite recording. In this way, the base editing output can also be normalized *post hoc* to the bacterial growth rate.

#### 3.1.3 Marketability and monetary considerations

The total manufacturing and operational cost of our pill design can be calculated by summing the total material, manufacturing and operational cost for each use of the pill.

The three-layer outer coating of the pill requires a 0.2694 mL solution consisting of Eudragit L100 dissolved in isopropanol and acetone, as described by Khan and Jialal, 2022. For the internal scaffolding, approximately 1,278 mm^3^ Type I bacterial cellulose is required for the construction of each pill. The sensing system is primarily composed of *E. coli* which after initial construction is indispensable and would not need to be factored into the cost. Using these metrics, the material cost per pill is approximately $0.427 USD.

According to well established next-generation sequencing platforms like Illumina, the operational costs of high throughput sequencing or targeted gene expression sequencing is approximately $23.00 USD per 150 bp, for a sequencing depth of 50, 000 copies. Thus, for our 400 bp sample, the approximate sequencing cost per pill is $61.33 USD.

It is difficult to present an exact measure of the labor and overhead costs associated with manufacturing the material, however, an educated estimate based on previous literature concerning similar products such as ingestible pills, biosensors, and other sequencing material would expect our metabolite biosensor cost to be around $90 USD - $100 USD per pill (Koklu et al., 2022). Therefore, the overall cost to produce our biosensor would be approximately $160 USD per pill.

## 4 Conclusion

The diagnostic process for MDD is currently guided by the DSM-V, a guide that is inherently limited in its strictly qualitative approach. Exploiting the GBA enables quantitative characterization of MDD via gut metabolites such as butyrate, indole, and tetrahydrofolate which are well supported in literature. In addition, we propose a rationale for the sensing of hydrogen peroxide and tetrathionate as indicators of MDD. We tested the feasibility of our proposed biosensor by prototype building of the sensors and kinetic modeling of the CRISPR-mediated reporter system. Modeling results demonstrate that the system achieves targeted sensing of metabolites within the large intestine, enabling robust recording of metabolite concentrations in a time- and dose-dependent manner. A *post hoc* normalization model successfully controlled for confounding due to variation in riboflavin concentrations, and produced a linear relationship between actual and predicted metabolite concentrations. Together, our study suggests a promising approach for a novel, potentially quantitative and objective assessment of MDD using an ingestible microbial biosensor.

## Data Availability

The original contributions presented in the study are included in the article/Supplementary Material, further inquiries can be directed to the corresponding authors.
