# Supplementary material for: The development of an ingestible biosensor for the characterization of gut metabolites related to major depressive disorder: hypothesis and theory
Source: Front Syst Biol. 2023 Dec 5;3:1274184. doi: 10.3389/fsysb.2023.1274184 (PMC12342046; doi:10.3389/fsysb.2023.1274184)
Supplement: Supplementary file 1 [file DataSheet1.docx]

Supplementary Material

# Supplementary Data.

No Supplementary Data

# Supplementary Figures and Tables

## Supplementary Figures


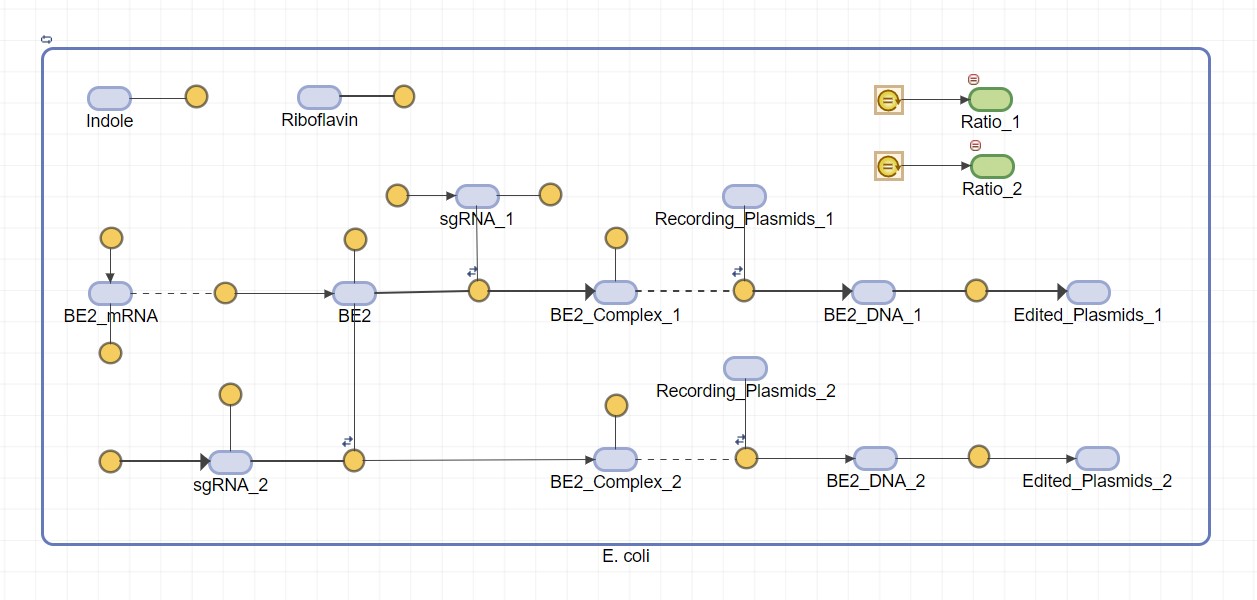


**Supplementary Figure 1.** Diagram of the overall plasmid editing system in SimBiology. Blue ovals represent chemical species, while yellow circles represent reactions.

**
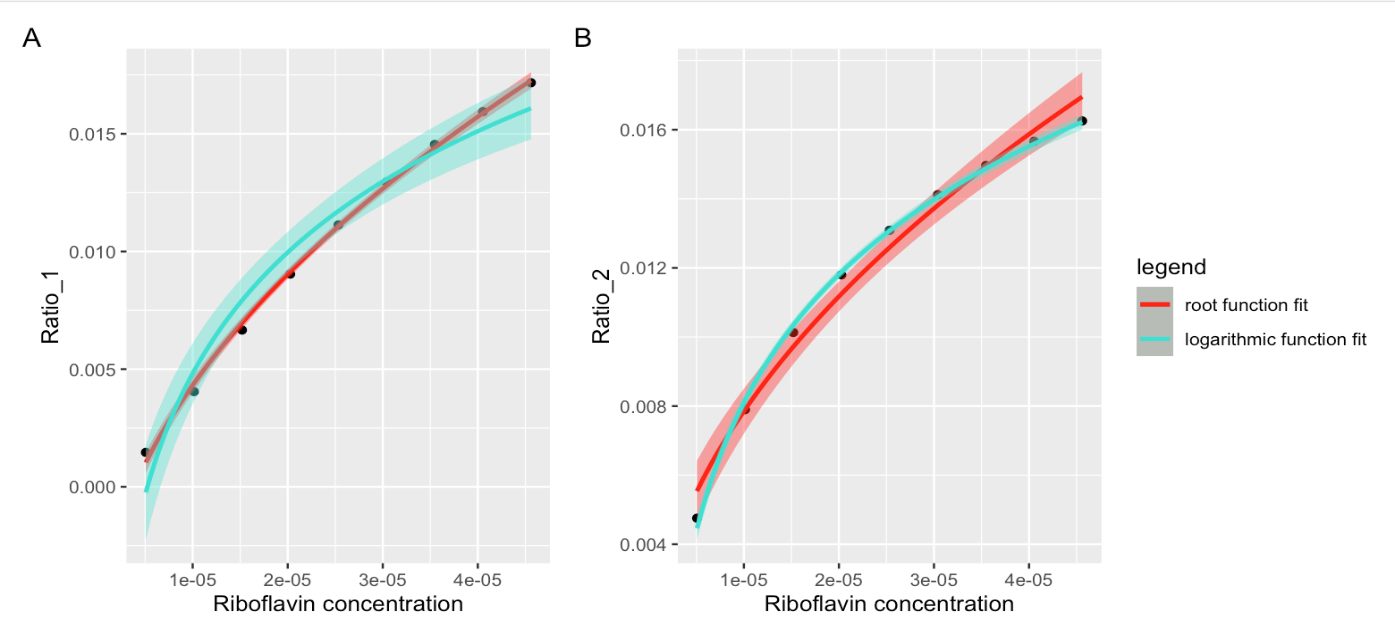
**

**Supplementary Figure 2.** Comparison of root vs logarithm relationships between variations in riboflavin concentration, and base-editing ratios at each editing site. Indole is constant.


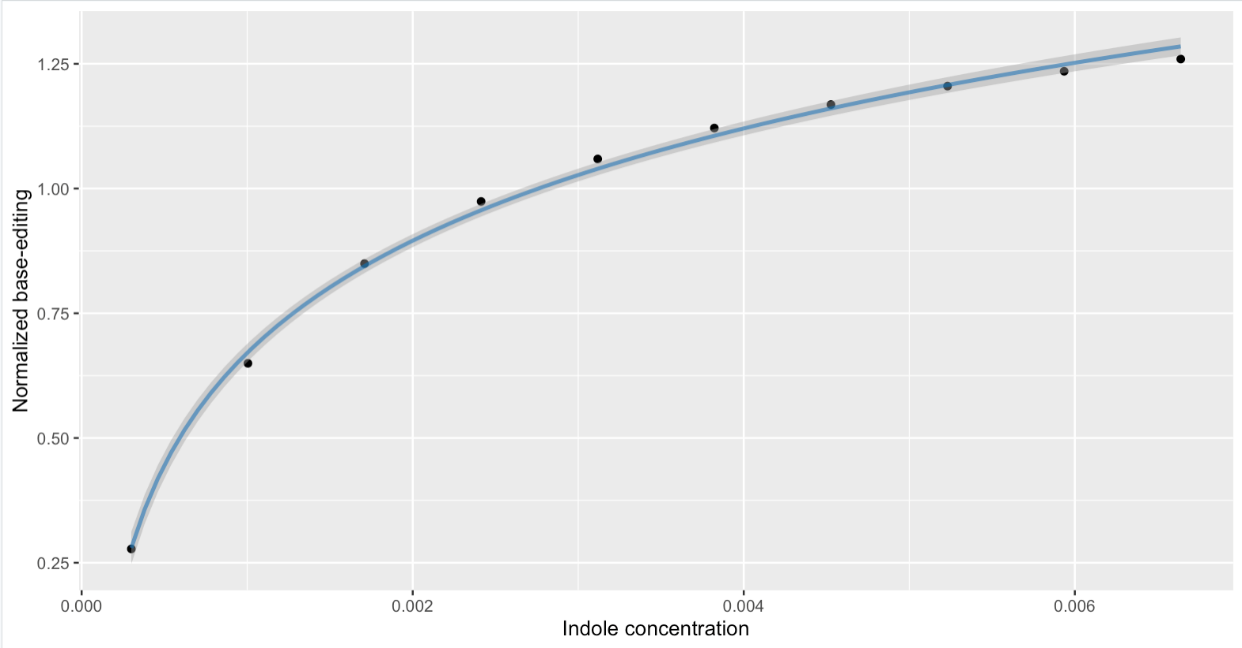


**Supplementary Figure 3.** Logistic relationship between normalized base-editing and actual indole concentration.

## Supplementary Tables

**Supplementary Table 1. Kinetic constants used in the model with specific assumptions that were made.**

| Category | Constant | Value | Source | Notes/Assumptions |
| --- | --- | --- | --- | --- |
| General constants | Volume of *E. coli* | 1.80×10^-15^ *L* | (Outten and O’Halloran, 2001) |  |
|  | Transcription rate | 45 *BP/(s·RNAP)* | (Yu et al., 2006) | Converted from *BP/(s·RNAP)* to *M/(s·RNAP)* by factoring in the gene lengths:  45 BPs RNAP1 mRNA# BP1 cell1.810-15 L1 mol6.0223 mRNA |
|  | Translation rate | 15 *AA/(s·ribosome)* | (Yu et al., 2006) | Converted from *AA/(s·ribosome)* to *1/(s·ribosome)* by factoring in the gene length:  15 AAs ribosome3 BP1 AA1# BP |
|  | mRNA degradation rate | 2.1×10^-3^ *s^-1^* | (Bakshi et al., 2012) |  |
|  | Protein degradation rate | 3.85×10^-5^ *s^-1^* | (Krzysztoń et al., 2019) | Assumed the same degradation rate for all proteins. Indole and riboflavin were approximated to have the same degradation rate as proteins as their degradation in *E. coli* is not well characterized. |
|  | sgRNA degradation rate | 2.1×10^-3^ *s^-1^* | (Bakshi et al., 2012) | Assumed that sgRNA behaves similarly to mRNA. |
|  | Number of RNA polymerases per cell | 1800 *RNAP* | (Bremer and Dennis, 2008) | Lower bound taken, and assumed steady state. |
|  | Number of ribosomes per cell | 8000 *ribosomes* |  |  |
| Gene lengths | BE2 gene | 5103 *BP* | (Tang and Liu, 2018) |  |
|  | sgRNA gene | 96 *BP* (76 for scaffold, 20 for spacer) | (Tang and Liu, 2018) |  |
| Riboflavin-  induced BE2 and sgRNA1 transcription | K (activation coefficient) | 20×10^-6^ *M* | (Liu et al., 2022) | Assumed K to be the concentration of riboflavin required for significant upregulation. |
| Indole-  induced sgRNA2 transcription | K (activation coefficient) | 1.347×10^-3^ *M* | (Matulis et al., 2022) |  |
| BE2-sgRNA dimerization | k_association_ | 6.1 *(s·M)^-1^* | (Raper et al., 2018) | Binding occurs very fast, so the rate is primarily related to the conformational change of Cas9 from apo to holo form. |
|  | k_dissociation_ | 5.50×10^-4^ *s^-1^* |  |  |
| BE2 complex-  plasmid dimerization | k_association_ | 3.00×10^6^ *(s·M)^-1^* |  | Lower bound taken since we assumed less ideal conditions. |
|  | k_dissociation_ | 1.3 *s^-1^* |  |  |
| Base editing | k_cat_ | 132 *s^-1^* | (Hall et al., 2011) |  |
|  | [recording plasmids]_0_ | 1.49×10^-9^ *M* | (Tang and Liu, 2018) | Calculated from presented values and assumed steady state. |

**Supplementary Table 2.** Constants for Normalization Model at t = 10 hours.

|  |  |  |
| --- | --- | --- |
| 1 | 3.606359 | -0.007085139 |
| 2 | 0.005352677 | 0.06972578 |
| 3 | 0.3244967 | 2.91213 |

**Supplementary Table 3.** Cost Analysis of Proposed Pill

| Amount of Material Required for Proposed Pill Design | | | | |
| --- | --- | --- | --- | --- |
| Material | Surface Area (mm^2^) | Thickness (mm) | Volume (mL) | Volume (mm^3^) |
| Eudragit® L100 solution (Enteric coating) | 2694 | 0.1 | 0.2694 | NA |
| Type-I  Bacterial Cellulose | 1278 | 1 | NA | 1278 |
| Solution composition of Enteric Coating (Eudragit® L100) | | | | |
| Material | Eudragit® L100 (g) | isopropanol and acetone (1 : 1) (mL) | Mass of Eudragit® L100 per 1 mL of solution (g/mL) | |
| Eudragit® L100 solution (Enteric coating) | 3 | 40 | 0.075 | |
| Material Cost of Proposed Pill Design | | | | |
| Material | Cost per 1 gram (USD/g) | Cost per mm^3^ (USD/mm^3^) | Volume (mL) | Cost per pill (USD) |
| Eudragit® L100 solution (Enteric coating) | 2.694 | NA | 0.2694 | 0.0008 |
| Type-I  Bacterial Cellulose | NA | 0.00033 | NA | 0.4260 |
| Operational Cost of Proposed Pill Design | | | | |
| Operation | Cost per 150 bp (USD) | | Cost per pill (USD) | |
| High Throughput Sensing (Target gene sequencing) | 23 | | 61.33 | |
